# Glycosyltransferases: glycoengineers in human milk oligosaccharide synthesis and manufacturing

**DOI:** 10.3389/fmolb.2025.1587602

**Published:** 2025-04-30

**Authors:** Alanna S. Slater, Andrew G. McDonald, Rita M. Hickey, Gavin P. Davey

**Affiliations:** ^1^ Teagasc Food Research Centre, Moorepark, Fermoy, Ireland; ^2^ School of Biochemistry and Immunology, Trinity College Dublin, Dublin, Ireland

**Keywords:** glycosyltransferases, sialyltransferase, fucosyltransferase, human milk oligosaccharides (HMOs), biosynthesis

## Abstract

Human milk oligosaccharides (HMOs) are a diverse group of complex carbohydrates that play crucial roles in infant health, promoting a beneficial gut microbiota, modulating immune responses, and protecting against pathogens. Central to the synthesis of HMOs are glycosyltransferases, a specialized class of enzymes that catalyse the transfer of sugar moieties to form the complex glycan structures characteristic of HMOs. This review provides an in-depth analysis of glycosyltransferases, beginning with their classification based on structural and functional characteristics. The catalytic activity of these enzymes is explored, highlighting the mechanisms by which they facilitate the precise addition of monosaccharides in HMO biosynthesis. Structural insights into glycosyltransferases are also discussed, shedding light on how their conformational features enable specific glycosidic bond formations. This review maps out the key biosynthetic pathways involved in HMO production, including the synthesis of lactose, and subsequent fucosylation and sialylation processes, all of which are intricately regulated by glycosyltransferases. Industrial methods for HMO synthesis, including chemical, enzymatic, and microbial approaches, are examined, emphasizing the role of glycosyltransferases in these processes. Finally, the review discusses future directions in glycosyltransferase research, particularly in enhancing the efficiency of HMO synthesis and developing advanced analytical techniques to better understand the structural complexity and biological functions of HMOs.

## 1 Introduction

Human milk oligosaccharides (HMOs) are critical components of breast milk, attracting significant attention for their vital roles in infant health and development. Central to the biosynthesis of HMOs is a family of enzymes known as glycosyltransferases, which are responsible for constructing the diverse glycan structures that define the bioactivity of HMOs. Glycosyltransferases orchestrate the precise arrangement of sugars, giving rise to the unique HMO structures that provide breastfed infants with numerous health benefits. The pivotal role of glycosyltransferases in HMO production is to control the sequential addition of sugars that generate the unique array of HMO structures found in breast milk. A comprehensive understanding of these enzymes is essential not only for elucidating natural HMO synthesis but also for enabling the replication of this process in external settings, such as laboratories and industrial-scale manufacturing and production.

The ability to synthesize HMOs artificially holds great potential, particularly for enhancing infant formulas, narrowing the nutritional gap between formula-fed and breastfed infants. Beyond infant nutrition, HMOs have been implicated in modulating the immune system, promoting gut health, and potentially even influencing cognitive development. Understanding HMO biosynthesis is not only essential for improving infant formula but also holds potential for broader applications in the fields of medicine and biotechnology. Synthetic HMO production opens new avenues for exploring their therapeutic potential in treating various health conditions and advancing biotechnological applications.

**FIGURE 1:**
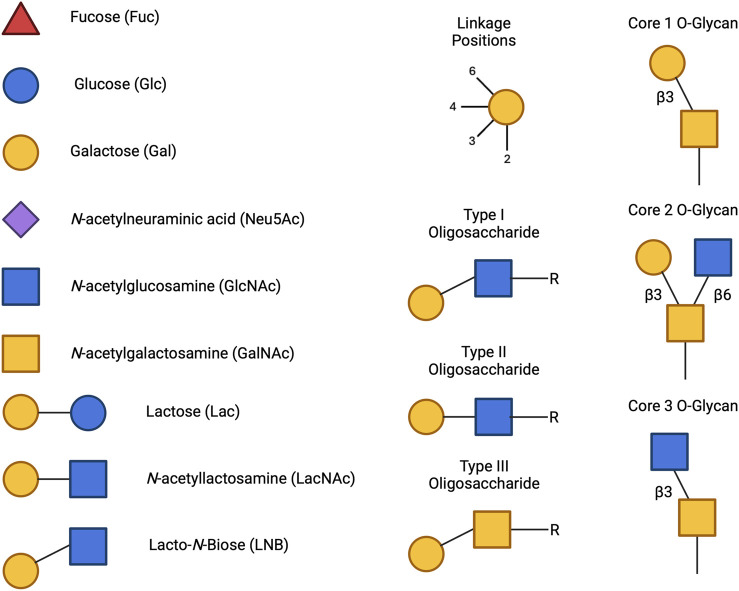
Structural Components of Human Milk Oligosaccharides: Schematic representation of human milk oligosaccharides (HMOs) and their structural components. The key monosaccharides in HMO synthesis include fucose (red triangle), glucose (blue circle), galactose (yellow circle), *N*-acetylglucosamine (blue square), and *N*-acetylneuraminic acid (purple diamond). Foundational HMO building blocks such as Lactose (Lac), *N*-acetyllactosamine (LacNAc) and Lacto-*N*-Biose (LNB) are also shown. The central panel depicts the type I, type II and type III oligosaccharide structures, distinguished by their linkage positions. The right panel presents the structural cores of *O*-glycans: Core 1, Core 2 and Core 3.

HMOs are a unique and complex group of non-digestible carbohydrates that constitute the third-largest solid component of human milk, after lactose and lipids (Zivkovic et al., 2011). Comprising over 200 structurally distinct molecules (Carr et al., 2021), HMOs are characterized by a core lactose or lacto-*N*-biose structure extended by various monosaccharide units, including glucose, fucose, sialic acid, galactose, and *N*-acetylglucosamine (Chen, 2015; Figure 1). This structural diversity underpins the wide array of biological functions attributed to HMOs, which are not present in significant quantities in the milk of other mammals, making them a defining feature of human breast milk.

The composition of HMOs in breast milk is highly variable, influenced by factors such as genetics (particularly the mother’s secretor status), stage of lactation, and environmental factors. “Secretor Positive” (Mulinge et al., 2024) and “Lewis Positive” (Blank et al., 2012) individuals express specific enzymes involved in HMO synthesis, impacting the types and quantities of HMOs produced (Table 1). Genetic variations in genes encoding glycosyltransferases and sialyltransferases influence HMO production levels and types. HMOs are broadly categorized into several groups based on their structural components, including neutral (core) HMOs (Wu et al., 2010), sialylated HMOs (Wu et al., 2011), and fucosylated HMOs (Orczyk-Pawiłowicz and Lis-Kuberka, 2020). Neutral HMOs, lacking a charge and neither acidic or basic, include examples such as Lacto-*N*-tetraose (LNT), Lacto-*N*-neotetraose (LNnT), and Lacto-*N*-fucopentaose IV (LNFP IV) (Table 2). Fucosylated HMOs characterized by a fucosyl group, include structures such as 2′-fucosyllactose (2′-FL) and 3-fucosyllactose (3-FL) (Table 3). Sialylated HMOs which are negatively charged at physiological pH, typically feature configurations such as 3′-sialyllactose (3′-SL) and 6′-sialyllactose (6′-SL) (Table 4).

**TABLE 1:** Classification of Lewis and Secretor phenotypes and their associated HMOs.

Classification	Se (FUT2)	Le (FUT3)	Fucose linkages	HMOs secreted
Lewis Positive Secretor Positive	+	+	α1-2 α1-3 α1-4	All HMOs
Lewis Positive Secretor Negative	–	+	α1-3 α1-4	3-FL LNFP-II LNFP-III
Lewis Negative Secretor Positive	+	–	α1-2 α1-3	2′-FL 3-FL LNFP-I LNFP-III
Lewis Negative Secretor Negative	–	–	α1-3	3-FL LNFP-III LNFP-V

**TABLE 2:** Names, Abbreviations, and Structures of a sample of Neutral HMOs.

Name	Abbreviation	Structure	References
Lacto-*N*-tetraose	LNT	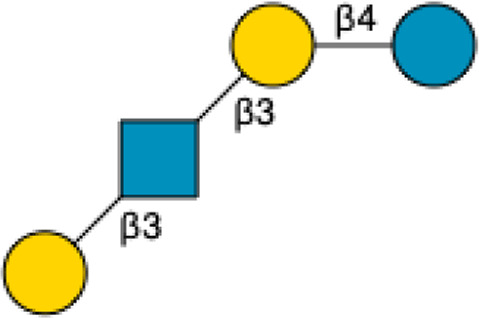	Chen (2015) McDonald et al. (2022)
Lacto-*N*-neotetraose	LNnT	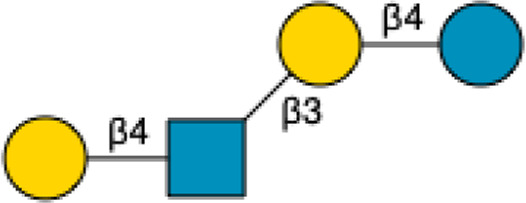	Chen (2015) McDonald et al. (2022)
Lacto-*N*-hexaose	LNH	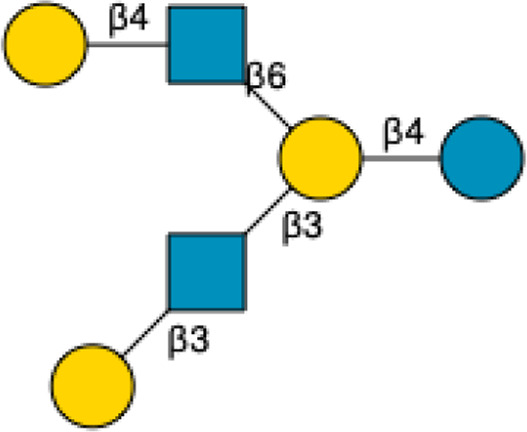	Chen (2015) McDonald et al. (2022)
Lacto-*N*-neohexaose	LNnH	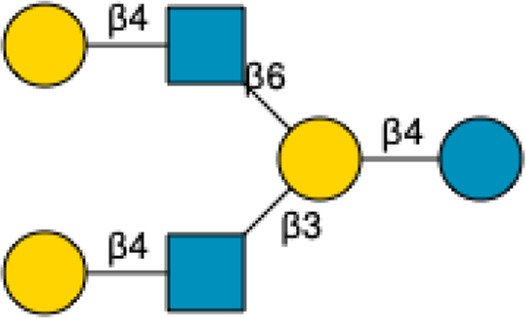	Chen (2015) McDonald et al. (2022)

**TABLE 3:** Names, Abbreviations, and Structures of a sample of Fucosylated HMOs.

Name	Abbreviation	Structure	References
2′-Fucosyllactose	2′-FL	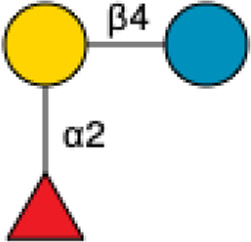	Chen (2015) McDonald et al. (2022)
3-Fucosyllactose	3-FL	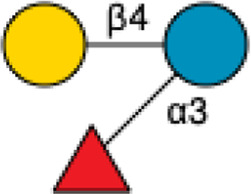	(Chen, 2015) McDonald et al. (2022)
Difucosyllactose	DFL	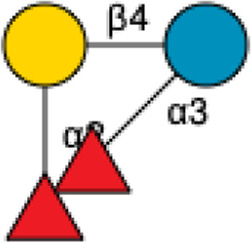	Chen (2015) McDonald et al. (2022)
Lacto-*N*-fucopentaose	LNFP-I	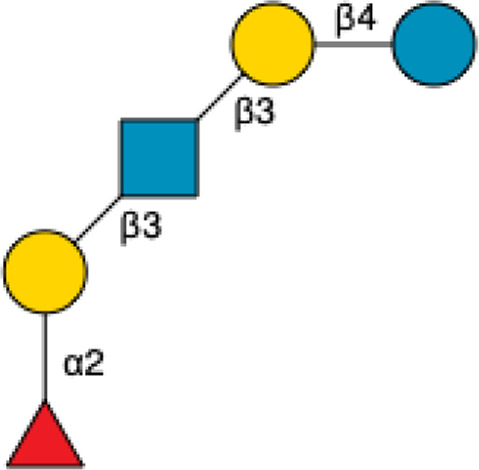	Chen (2015) McDonald et al. (2022)
	LNFP-II	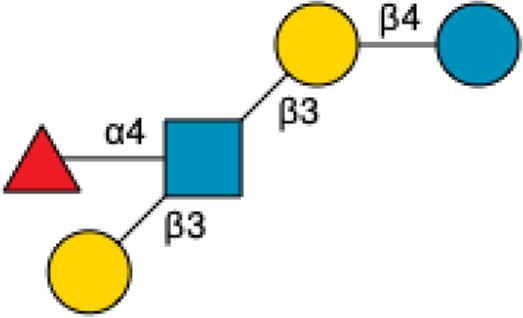	Chen (2015) McDonald et al. (2022)
	LNFP-III	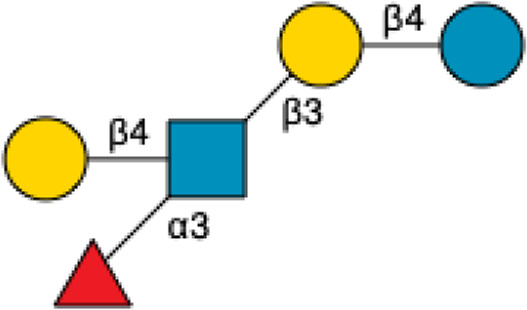	Chen (2015) McDonald et al. (2022)
	LNFP-V	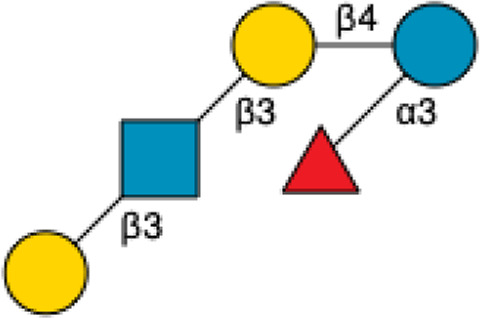	Chen (2015) McDonald et al. (2022)
	LNFP-VI	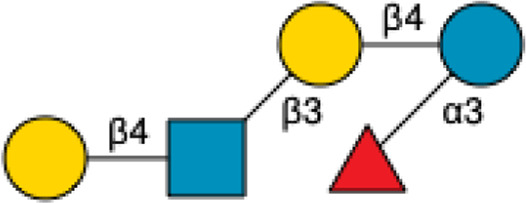	Chen (2015) McDonald et al. (2022)
Difucosylsialyllacto-*N*-hexaose^a^	DFS-LNH-I	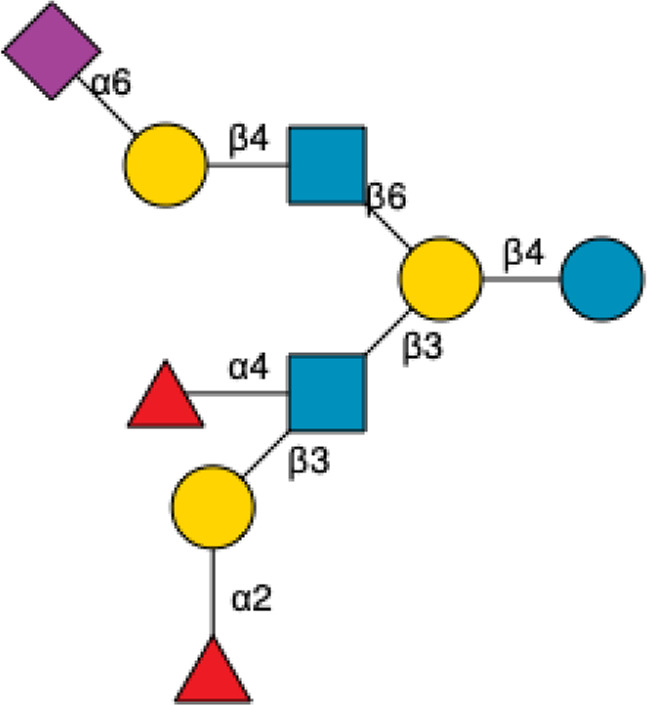	Chen (2015) McDonald et al. (2022)

^a^ Both fucosylated and sialylated.

**TABLE 4:** Names, Abbreviations, and Structures of a sample of Sialylated HMOs.

Name	Abbreviation	Structure	References
3′-Sialyllactose	3′-SL	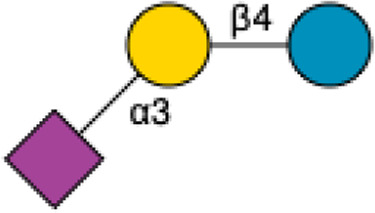	Chen (2015) McDonald et al. (2022)
6′-Sialyllactose	6′-SL	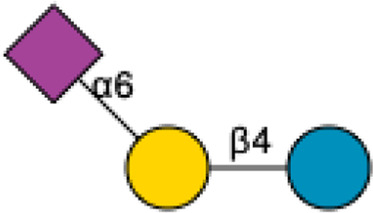	Chen (2015) McDonald et al. (2022)
Sialyllacto-*N*-tetraose	LST a	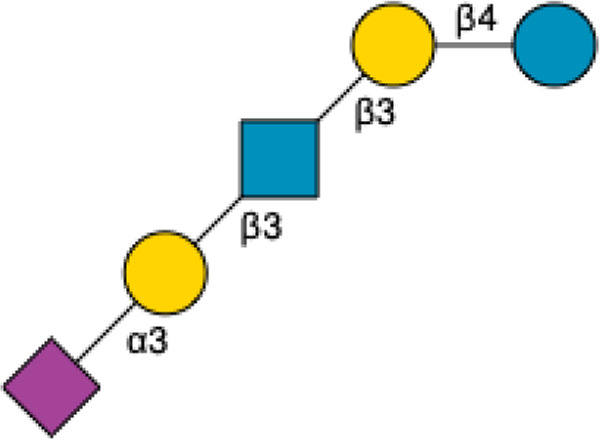	Chen (2015) McDonald et al. (2022)
	LST b	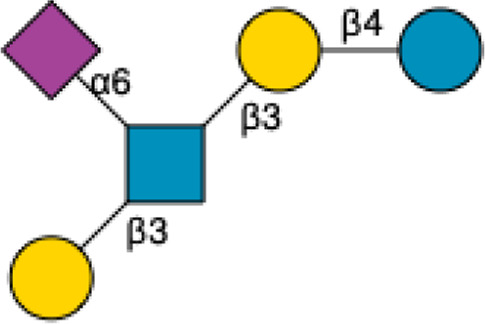	Chen (2015) McDonald et al. (2022)
	LST c	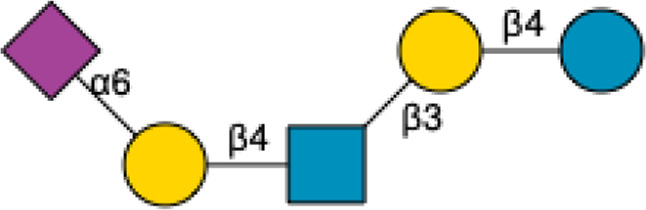	Chen (2015) McDonald et al. (2022)
Disialyllacto-*N*-tetraose	DSLNT	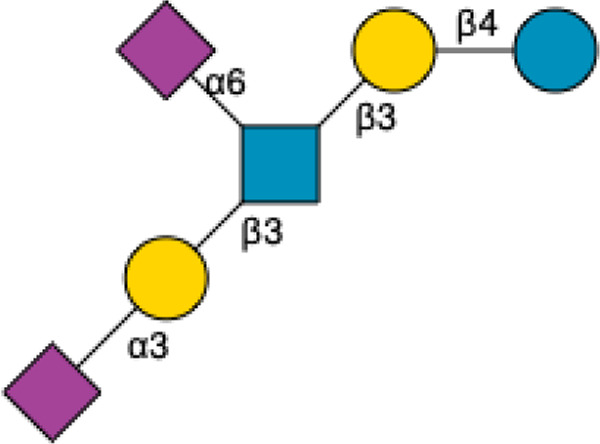	Chen (2015) McDonald et al. (2022)
Fucosylsialyllacto*-N-*neohexaose^a^	FS-LNnH-I	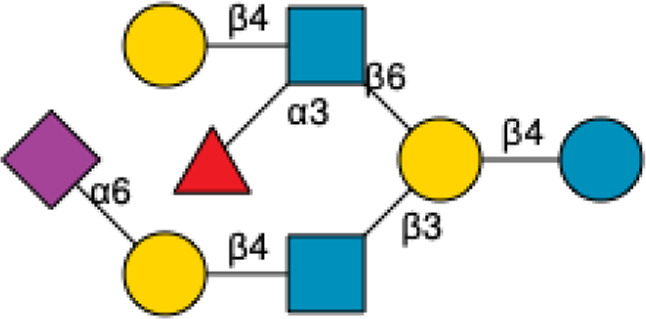	Chen (2015) McDonald et al. (2022)

^a^ Both fucosylated and sialylated.

The health implications of HMOs are extensive, driven by their ability to influence the infant gut microbiome and immune system. One of the most well-documented functions of HMOs is their prebiotic effect, selectively promoting the growth of beneficial gut bacteria, particularly bifidobacterium species (Okburan and Kızıler, 2023). These bacteria metabolize HMOs, producing short-chain fatty acids and other metabolites that contribute to a healthy gut environment. This microbial fermentation is crucial for protecting against pathogenic bacteria, reducing the risk of infections, and supporting the maturation of the infant’s immune system. In addition to their prebiotic role, HMOs serve as decoy receptors that prevent pathogens from binding to the intestinal mucosa (Craft and Townsend, 2018), thereby reducing the risk of infections such as diarrhoeal diseases and respiratory tract infections. For instance, 2′-FL has been shown to inhibit the binding of certain strains of *Escherichia coli* (Manthey et al., 2014) and *Campylobacter jejuni* (Morrow et al., 2004; Ruiz-Palacios et al., 2003) to the gut epithelium, lowering the incidence of gastrointestinal infections in infants. HMOs also contribute to immune system development by modulating immune cell responses (Plaza-Díaz et al., 2018). Studies have shown that HMOs can influence the expression of inflammatory cytokines and enhance the maturation of immune cells, contributing to a balanced immune response (Slater et al., 2024). Additionally, sialylated HMOs have been implicated in neurodevelopment (Fan et al., 2023), with evidence suggesting that they play a role in cognitive function by supporting brain development and synaptic connectivity.

Beyond infancy, the health benefits of HMOs are being explored in various contexts, including their potential therapeutic applications for adults. HMOs have been investigated for their role in managing conditions such as irritable bowel syndrome (IBS) (Iribarren et al., 2020; Palsson et al., 2020), where they may help restore a healthy gut microbiota, and in immune-related disorders, where their anti-inflammatory properties could be beneficial. Ultimately the diverse composition of HMOs and their wide-ranging health implications highlight their significance in human milk and beyond. The ability to replicate these complex molecules for inclusion in infant formula and other therapeutic products has the potential to enhance health outcomes across different populations. Understanding the intricate relationship between HMO structure and function is therefore critical for advancing both scientific knowledge and practical applications in nutrition and medicine.

In this review we focus on the pioneering role of glycosyltransferases in HMO synthesis and their significance in commercial production. By exploring the molecular mechanisms behind HMO biosynthesis and the enzymes that drive these processes, we aim to provide insights into the development of innovative strategies for large-scale HMO production. We highlight the crucial role of glycosyltransferases in promoting HMO diversity and functionality, which is key for synthetic HMO production. This diversity contributes to improved infant health, though our emphasis remains on the significance of glycosyltransferases in HMO biosynthesis.

## 2 Glycosyltransferases

Glycosyltransferases are a superfamily of enzymes that catalyse the transfer of sugar molecules from a donor, usually a nucleotide sugar, to an acceptor molecule, such as a protein, lipid, or another sugar (Lairson et al., 2008; Zhao et al., 2023). This process is responsible for the biosynthesis of polysaccharides, disaccharides, glycoconjugates (including glycoproteins, glycolipids, and proteoglycans) and oligosaccharides, a critical step in the intricate assembly of HMOs. Glycosylation is an essential modification of biomolecules that affects their structure, stability, solubility, and function.

### 2.1 Classification of glycosyltransferases

Glycosyltransferases are transmembrane type-II proteins that are mainly present in the Endoplasmic Reticulum (ER) and Golgi apparatus (Paulson and Colley, 1989; Kleene and Berger, 1993). These enzymes can present as GT-A (Taujale et al., 2020), GT-B (Albesa-Jové et al., 2014) or more recently characterised, GT-C (Alexander and Locher, 2023) fold. Many families of glycosyltransferases have been identified based on their sequence, structural similarities while some are separated based on the type of nucleotide sugar involved (i.e., sialyltransferases transfer sialic acid using CMP-Neu5Ac as the donor). There are currently 116 different glycosyltransferase families identified according to CAZy (Coutinho et al., 2003; Drula et al., 2022) (GT1-GT116). Each family has a specific set of substrates and catalytic mechanisms, and certain families have been further divided into subfamilies based on their phylogenetic relationships and substrate specificities. As of 2024 there were 215 GT structures characterised (GT-A: 125, GT-B: 53, GT-C: 25, other: 12 (Yagi et al., 2024). Characterizing these enzymes has proven challenging due to the difficulty in obtaining glycosyltransferases in sufficient quantities for crystallographic studies, while their larger size makes them unsuitable for analysis by NMR. Additionally, the number of newly determined glycosyltransferase structures has significantly decreased in recent years, despite an increase in non-redundant glycosyltransferase sequences due to the growing availability of genomic and metagenomic data. This indicates that, while sequence information continues to expand, structural insights into glycosyltransferases have not kept pace (Gloster, 2014).

Nucleotide·sugar+Acceptor−GT→Acceptor·sugar+Nucleotide

GT-A glycosyltransferase enzymes have a single-domain architecture characterized by a central β-sheet surrounded by α-helices (Paulson and Colley, 1989; Kleene and Berger, 1993). The GT-A fold typically contains a Rossmann-like fold (Breton et al., 2012), which is a common protein fold found in many enzymes, including dehydrogenases, kinases, and synthases. The active site of GT-A enzymes usually have a conserved DXD motif (aspartate-any residue-aspartate) which coordinates the divalent cations (usually Mn^2+^ or Mg^2+^) required for their activity (Taujale et al., 2020). GT-A glycosyltransferases are involved in transferring sugars from nucleotide sugar donors (e.g., UDP-, GDP-) to acceptors, which can be proteins, lipids, or other sugars. The catalytic mechanism of GT-A enzymes involves an inverting reaction, where the donor sugar is transferred to the acceptor with inversion of configuration at the anomeric carbon (Moremen and Haltiwanger, 2019a; Bandi et al., 2020). Sialyltransferases from the GT29 subfamily, such as CMP*-N-*acetylneuraminate-β-galactosamide-α-2,3-sialyltransferase 1 (ST3GAL1) and ST6GAL1, are examples of GT-A enzymes (Moremen and Haltiwanger, 2019b).

GT-B glycosyltransferases feature a bi-lobal architecture, where two β/α/β domains are connected by a flexible linker where the active site is located (Albesa-Jové et al., 2014). This structural arrangement allows the enzyme to undergo conformational changes that facilitate substrate binding and catalysis. GT-B enzymes typically perform a retaining mechanism, preserving the stereochemistry of the sugar donor during transfer. Some fucosyltransferases, which add fucose residues to HMOs, belong to this class including α1,2-fucosyltransferases 2 (FUT2) and α1,2-fucosyltransferases 3 (FUT3) (Albesa-Jové et al., 2014). Other examples of GT-B glycosyltransferases include glycogen synthase (GLGA) and UDP-galactopyranose mutase (UGM). The GT-B fold lacks the conserved DXD motif found in GT-A. Instead, GT-B enzymes often has other conserved motifs critical for binding and catalysis and does not require Mn^2+^ or Mg^2+^ for their enzymatic activity (Lairson et al., 2008). GT-B glycosyltransferases also use nucleotide sugar donors but tend to be more diverse in the types of acceptor molecules they can modify. This is due to their two-domain architecture, which forms a deep and often flexible cleft between the N- and C-terminal domains. This structural arrangement allows for more variability in the shape, size, and chemistry of the acceptor-binding site, enabling accommodation of a broader range of substrates such as small molecules, peptides, lipids, or even unusual glycans (Lairson et al., 2008). In contrast, GT-A enzymes often have a more rigid and conserved acceptor-binding pocket that requires donor binding for its formation and is tailored for specific glycan or protein substrates (Chang et al., 2011).

GT-C glycosyltransferase enzymes are more recently characterised and are membrane-bound with a distinct topology with multiple transmembrane helices. Until very recently, their structure was less well characterized compared to GT-A and GT-B, owing to the challenges in crystallizing membrane proteins (Lizak et al., 2011). The active sites of GT-C enzymes are located within the membrane or at the membrane interface. GT-C enzymes do not use nucleotide sugars as donors and often utilise a lipid-linked sugar donor, such as dolichol-phosphate-mannose, in their catalytic processes (Alexander and Locher, 2023; Bai and Li, 2021). While GT-C enzymes are less prominent in HMO biosynthesis, their role in glycoprotein and glycolipid biosynthesis highlights the diversity of glycosyltransferase function across different biological systems. These enzymes are multi-pass transmembrane proteins localized in the endoplasmic reticulum (ER) and primarily utilize lipid-linked sugar donors, making them more suited to protein glycosylation (Bai and Li, 2021) rather than the sequential addition of nucleotide sugars to free oligosaccharide chains. Compared to GT-A and GT-B glycosyltransferases which localise to the Golgi and utilise nucleotide sugars, as the Golgi apparatus, unlike the ER, contain many sugar nucleotides (Berninsone and Hirschberg, 2000). GT-C glycosyltransferases are often involved in the synthesis of polysaccharides or glycoproteins in membranes (Bohl et al., 2021), such as in the construction of bacterial cell walls or the glycosylation of proteins in the ER. Examples of GT-C glycosyltransferases include *N*-glycosyltransferases STT3A and STT3B (Alexander and Locher, 2023).

### 2.2 Catalytic activity of glycosyltransferases

The active site of the enzyme is formed by residues from multiple domains, which orient the donor and acceptor molecules for the transfer reaction. Several glycosyltransferases operate through an ordered sequential Bi-Bi kinetic mechanism, in which the enzyme first binds the donor substrate before interacting with the acceptor substrate (Lairson et al., 2008). The catalytic mechanism of glycosyltransferases involves the formation of a glycosyl-enzyme intermediate, in which the sugar molecule is covalently attached to a catalytic residue, typically a serine, threonine or cysteine. The intermediate is then attacked by the acceptor substrate, leading to the transfer of the sugar moiety and the release of the enzyme (Rini et al., 2009), with the product dissociating from the enzyme prior to its associated nucleotide sugar (Lairson et al., 2008). The reaction is highly specific and requires the correct alignment of the donor and acceptor molecules and also requires stabilisation by intermediates such as hydrogen bonds. The variable domain of glycosyltransferases can adopt different folds and orientations, depending on the substrate and the catalytic mechanism (Breton et al., 2006). Some glycosyltransferases have a single domain, while others have multiple domains that can interact with each other or with other proteins such as β-1,4-Galactosyltransferase 1 (B4GALT1), which has the capacity to induce a conformational change to itself once bound to α-lactalbumin (LALB) and also has the capacity to bind glucose, GlcNAc and UDP-galactose (Rini et al., 2009). In its default state, it transfers galactose to GlcNAc, with an open substrate-binding site and an exposed hydrophobic *N*-acetyl group-binding pocket. Upon binding to LALB, B4GALT1 closes the *N*-acetyl pocket to optimise glucose binding, allowing for lactose synthesis to occur. Glycosyltransferases can have one or more catalytic domains, and can also have non-catalytic domains that modulate their activity, localization or specificity. Most glycosyltransferases have transmembrane domains that anchor them to the Golgi or endoplasmic reticulum membranes, where they can catalyse the synthesis of glycolipids or glycoproteins (Maccioni, 2007). Other glycosyltransferases have domains that recognize specific protein motifs or glycan structures, allowing them to modify only certain substrates.

Glycosyltransferases exhibit a strong preference for their cognate nucleotide sugar donors, dictated by enzyme-substrate interactions at the nucleotide-binding pocket. For example, work by Thorson et al. on glycosyltransferase-catalysed reactions showed how the leaving group ability of sugar donors influences the production of desired sugar nucleotides and that simple donor modifications can shift reaction equilibria, influencing substrate selectivity (Gantt et al., 2011). Further work by this group demonstrated that modifying nucleotidyltransferase RmlA by directed evolution expanded its nucleotide and sugar 1-phosphate promiscuity, highlighting the potential for engineering glycosyltransferase specificity (Moretti et al., 2011). Each glycosyltransferase is responsible for one linkage in oligosaccharide formation such that fucosyltransferases add fucose, sialyltransferases add sialic acid, etc (Coutinho et al., 2003). McDonald et al. investigated a system where multiple acceptor substrates compete for a single glycosyltransferase enzyme. The authors identified that high concentrations of an acceptor substrate can lead to the formation of abortive complexes, resulting in non-Michaelian kinetics. This phenomenon can cause bistability in open systems, where the system can reside in two distinct stable states under the same conditions (McDonald et al., 2018). This aligns with studies showing that some glycosyltransferases form higher-order oligomers, which influence catalytic efficiency, substrate specificity, and sequential glycosylation by altering enzyme conformation or facilitating substrate channelling. Together, these mechanisms highlight the dynamic regulation of glycosylation and its potential for pathological disruption (Varki et al., 2022).

The activity of glycosyltransferases is regulated by several mechanisms, including substrate availability, enzyme localization, post-translational modifications (PTMs), and protein-protein interactions. For instance, certain glycosyltransferases, e.g., FUT2, require specific chaperones such as UGT1A1, which aid in their proper folding and activity (Ju and Cummings, 2002). Yet other glycosyltransferases such as B4GALT1 require co-factors such as Mn^2+^ which acts as a metal ion that stabilizes the interaction between the enzyme and its nucleotide sugar donor, facilitating the transfer of galactose to the acceptor substrate (Unligil and Rini, 2000). Other glycosyltransferases are regulated by feedback inhibition (e.g., α-1,3-mannosyl-glycoprotein 2-β*-N-*acetylglucosaminyltransferase (GNT3)) (Voss, 2024) or proteolysis (e.g., Protein O-Fucosyltransferase 1 (POFUT1)) (Stanley and Okajima, 2010; Kopan and Ilagan, 2009). The expression of certain glycosyltransferases, such as B4GALT1, is regulated by hormones, such as prolactin and estrogen (Choi et al., 2012), which promote the differentiation and proliferation of mammary epithelial cells (MECs). These hormones enhance the expression of B4GALT1, the key enzyme responsible for lactose synthesis.

Glycosyltransferases can also show different substrate specificities and preferences, depending on the structure and sequence of their acceptor substrates and the availability of nucleotide sugars. Therefore, the availability of nucleotide sugars is a critical factor in HMO biosynthesis. The biosynthesis of nucleotide sugars themselves requires several enzymes and metabolic pathways, including the hexosamine biosynthesis pathway, the pentose phosphate pathway (PPP), and the salvage pathway (Mikkola, 2020). Thus, the availability of these pathways can affect the biosynthesis of nucleotide sugars and, consequently, the biosynthesis of HMOs. The catalytic activity of glycosyltransferases is tightly regulated by intracellular metabolic fluxes, governing nucleotide sugar biosynthesis, energy metabolism and precursor availability, all of which are crucial for efficient HMO synthesis. For example, fluxes through glycolysis, the pentose phosphate pathway (PPP) and nucleotide sugar interconversion pathways directly impact the pool of donor substrates required for glycosylation. Synergizing metabolic flux analysis with nucleotide sugar metabolism has provided valuable insights into how these factors influence glycosyltransferase-mediated glycosylation, as demonstrated in recombinant protein production using CHO cells (Burleigh et al., 2011). Additionally, understanding how metabolic fluxes regulate nucleotide sugar availability and enzyme activity offers a deeper perspective on how glycosyltransferase activity can be modulated to enhance glycosylation efficiency and specificity in engineered systems (McDonald et al., 2016a). The composition of the lactating mammary gland can also affect the biosynthesis of HMOs. Several factors, such as maternal diet (Azad et al., 2018), genetics (Kumazaki and Yoshida, 1984; Johnson and Watkins, 1992), and environmental factors (Azad et al., 2018), can influence the expression and activity of glycosyltransferases and the availability of nucleotide sugars. These factors can affect the structure and diversity of HMOs and, consequently, their biological activity.

The understanding of the intricate mechanisms employed by glycosyltransferases not only enriches the field of HMO research but also holds immense potential for the artificial production of these oligosaccharides. By capitalizing on this understanding, we can strive towards the synthetic reproduction of HMOs, thereby unveiling promising pathways for their integration into infant formula and a wide range of other applications.

### 2.3 Structural insights into glycosyltransferases

Structural characterization of glycosyltransferases involved in HMO biosynthesis represents a cornerstone in understanding the molecular mechanisms underlying the synthesis of these complex oligosaccharides. Through employing techniques such as X-ray crystallography (XRC) and nuclear magnetic resonance (NMR) spectroscopy, researchers have been able to elucidate the three-dimensional (3D) structures of glycosyltransferases at atomic resolution (Nagae et al., 2020; Peters et al., 2017; Gagnon et al., 2017). Complementing these studies, molecular modelling and simulation studies, such as molecular dynamics simulations and quantum mechanics/molecular mechanics (QM/MM) calculations, have provided valuable insights into the conformational changes and molecular dynamics occurring during enzyme-substrate binding and catalysis (Taujale et al., 2021a; Imberty et al., 2006; Heissigerova et al., 2003). Another key area of focus in structural biology and computational modelling has been the elucidation of the structural determinants of glycosyltransferase specificity and catalytic activity. By comparing the structures of glycosyltransferases with different substrate specificities and catalytic efficiencies, researchers have identified specific amino acid residues and structural motifs that contribute to substrate recognition and binding (Kapitonov and Yu, 1999). For example, one study which used site-directed mutagenesis to identify the galactosyltransferase binding sites for UDP-galactose showed Phe305, Pro306, Asn307, and Asn308 in these galactosyltransferases are likely critical for enzyme catalysis or are positioned near the UDP-galactose binding site, but do not participate in manganese binding (Zu et al., 1995). Further mutational studies on ST3GAL1 revealed specific residues His299, Tyr300, and His316 are essential for enzyme activity (Jeanneau et al., 2004). Furthermore, site-directed mutagenesis studies have enabled the manipulation of glycosyltransferase activity by altering key amino acid residues within the enzyme. For instance, site-directed mutagenesis of the *B4GALT1* cytoplasmic domain revealed that serine and threonine residues are essential for cell surface expression and function (Hathaway et al., 2003). Substitution of these residues with aspartic acid reduced expression and function. These efforts have provided valuable insights into the molecular basis of glycosyltransferase function and have paved the way for the rational design of enzymes with enhanced catalytic properties for HMO synthesis.

Glycosyltransferases exhibit strict substrate specificity, which is both an advantage and a limitation. While specificity ensures the production of precise glycan structures, it also restricts the range of substrates that can be used. Characterizing the substrate specificity and catalytic mechanisms of glycosyltransferases is complex, requiring detailed structural and kinetic analyses. The structural complexity of glycosyltransferases, including their active sites and substrate-binding regions, poses a challenge for engineering efforts. High-resolution techniques such as X-ray crystallography and cryo-electron microscopy are necessary to elucidate enzyme structures, but these methods can be time-consuming and expensive. Glycosyltransferases exhibit low stability and solubility under industrial conditions such as elevated temperatures, extreme pH levels, and the presence of organic solvents—due to their complex folding requirements and potential reliance on cofactors or membrane associations. These harsh conditions can lead to misfolding, enzyme denaturation or aggregation, thereby reducing catalytic efficiency. This limits their practical application in large-scale HMO synthesis. Engineering enzymes for enhanced stability and solubility often requires extensive mutagenesis and screening, complicating the development process. Removing or modifying membrane anchor regions, optimizing codon usage, fusing solubility-enhancing tags could improve the solubility of these enzymes to facilitate selective binding to a resin like nickel-NTA or antibody. This is usually followed by size-exclusion chromatography or ion-exchange chromatography to improve purity.

## 3 Biosynthesis pathways of human milk oligosaccharides

The intricate biosynthesis pathways of HMOs are orchestrated by multiple glycosyltransferase reactions. Table 5 outlines the glycosyltransferases involved in HMO biosynthesis along with their associated Enzyme Commission (EC) numbers, compiled from KEGG and UniProt databases (Table 5). Numerous types of glycosyltransferases are involved in the stepwise construction of HMOs, each adding specific sugars in a defined sequence (Zhao et al., 2023), which ultimately results in the complex structural variety characteristic of HMOs (Urashima et al., 2018; Table 6). This sequence-specific action not only ensures the correct architecture of HMOs but also influences their functionality and bioavailability in infant nutrition. An in-depth appreciation of these biosynthetic routes is indispensable for advancing the technology to synthesize HMOs extrinsically, thereby enriching infant formulae with these crucial biochemical constituents. Consequently, this knowledge paves the way for the industrial replication of HMOs, emulating the nuanced biochemical glycan milieu that is naturally present in human milk.

**TABLE 5:** Human GTs EC numbers KEGG database.

EC number	Gene name(s)
EC 2.4.1.22	LALBA; B4GALT1-3
EC 2.4.1.38	B4GALT1, B4GALT2, B4GALT3
EC 2.4.1.62	B3GALT4
EC 2.4.1.65	FUT3, FUT5
EC 2.4.1.68	FUT8
EC 2.4.1.69	FUT2
EC 2.4.1.86	B3GALT1, B3GALT2
EC 2.4.1.90	B4GALT1, B4GALT2, B4GALT4
EC 2.4.1.102	GCNT1
EC 2.4.1.133	B4GALT7
EC 2.4.1.134	B3GALT6
EC 2.4.1.146	B3GNT3
EC 2.4.1.147	B3GNT6
EC 2.4.1.149	B3GNT2, B3GNT3, B3GNT4
EC 2.4.1.150	GCNT2
EC 2.4.1.152	FUT4, FUT6
EC 2.4.1.206	B3GNT5
EC 2.4.1.274	B4GALT5, B4GALT6
EC 2.4.1.344	FUT1, FUT2
EC 2.4.3.1	ST6GAL2
EC 2.4.3.2	ST3GAL4
EC 2.4.3.3	ST6GALNAC1, ST6GALNAC2
EC 2.4.3.4	ST3GAL1, ST3GAL2
EC 2.4.3.6	ST3GAL3, ST3GAL6
EC 2.4.3.7	ST6GALNAC3, ST6GALNAC4
EC 2.4.3.9	ST3GAL5
EC 2.4.3.10	No assignment

**TABLE 6:** Key enzymes involved in human milk oligosaccharide synthesis - substrates, products, and functional insights.

Enzyme name	Substrate (donor)	Substrate (acceptor)	Product	HMOs produced	Additional notes	Refs
Lactose Synthase	UDP-Galactose	Glucose	Lactose	Base unit for HMOs	First enzyme in HMO synthesis, forming lactose	Brew et al. (1968)
B3GALT1	UDP-Galactose	GlcNAc-containing acceptors	Lacto*-N-*Biose (LNB)	Base unit for HMOs	First enzyme in HMO synthesis, forming LNB.	Hennet et al. (1998b)
B4GALT1	UDP-Galactose	GlcNAc-containing acceptors	Galβ1-4 GlcNAc (Lactosamine unit)	LNnT	Adds galactose to form precursors of linear and branched HMOs	Powell and Brew (1975) Hennet (2002)
B3GNT2	UDP-GlcNAc	Galβ1-4Glc(NAc)	GlcNAcβ1-3Galβ1-4Glc(NAc)	LNTri II	First enzyme in HMO synthesis, forming LNTri II	Dabrowski et al. (1983) Hosomi and Takeya (1989)
FUT2	GDP-Fucose	Terminal Gal residues	α1-2-fucosylated glycans	2′-FL	FUT2 activity determines the secretor status in humans	He and Liu (2014)
FUT3	GDP-Fucose	Galβ1-4GlcNAc residues	α1-3/α1-4-fucosylated glycans	LNFP I	FUT3 is key for Lewis antigen synthesis in HMOs	Martin et al. (1997)
FUT4	GDP-Fucose	Galβ1-4GlcNAc residues	α1-3-fucosylated glycans	LNFP structures	Redundant with FUT3 in some contexts	Martin et al. (1997)
FUT5	GDP-Fucose	GlcNAc or Gal residues	α1-3/α1-4-fucosylated structures	Extended HMOs with α1-3/α1-4 linkages	Activity overlaps with FUT3 and FUT4	Daniels et al. (2013)
FUT6	GDP-Fucose	GlcNAc or Gal residues	α1-3/α1-4-fucosylated glycans	Similar to FUT3, FUT4, FUT5	Key in forming Lewis X antigens	Daniels et al. (2013)
ST6GAL NAC1	CMP-Neu5Ac	GalNAc residues of O-glycans	α2-6-sialylated glycans	Sialyllacto*-N-*tetraose (LSTa)	Catalyses sialylation of O-glycan cores	Pei et al. (2024)
ST6GAL NAC2	CMP-Neu5Ac	GalNAc-Ser/Thr or Galβ1,3GalNAc	α2-6-sialylated glycans	DSLNT	Adds α2-6-linked sialic acid to extended HMOs	Pei et al. (2024)
ST3GAL1	CMP-Neu5Ac	Galβ1-3 GalNAc residues	α2-3-sialylated glycans	Sialyllacto*-N-*neotetraose (LSTc)	Initiates α2-3 sialylation of precursor HMOs	Kono et al. (1997)
ST3GAL3	CMP-Neu5Ac	Galβ1-3/β1-4Gal residues	α2-3-sialylated glycans	Sialylated branched and linear HMOs	Plays a major role in branching and extending HMOs	Kono et al. (1997)
ST3GAL4	CMP-Neu5Ac	Galβ1-4 GlcNAc residues	α2-3-sialylated glycans	Branched sialylated HMOs	Works in conjunction with FUT enzymes	Kono et al. (1997)

### 3.1 Lactose synthesis pathway

The biosynthesis of HMOs begins with the synthesis of lactose. Lactose, the predominant sugar in human milk, is synthesized from free glucose and UDP-galactose (Fassio et al., 2018) bound by a β1,4 glycosidic bond (Brew et al., 1968). This process occurs in the Golgi apparatus of mammary epithelial cells (MECs). The enzyme lactose synthase (LS; EC 2.4.1.22) is composed of two subunits A: β1,4-galactosyltransferase (encoded by the *B4GALT1* gene; EC 2.4.1.38) and B: α-lactalbumin (encoded by the *LALBA* gene). Together this enzyme complex catalyses the transfer of galactose from UDP-galactose to glucose, forming lactose in the trans-Golgi of MECs. Lactose expression in mammary epithelium is tightly controlled by key hormones such as prolactin, insulin, hydrocortisone (Turkington et al., 1968), and glucocorticoid (Sadovnikova et al., 2022). Beginning in early pregnancy, lactose levels peak *postpartum* as placental steroids decrease and lactogenic hormones rise, especially prolactin which is stimulated by suckling. Light exposure (Lim et al., 2021) (i.e., time of day) and sexual hormones (Jin et al., 2024) also affect milk production. The role of LS in milk production is debated, but LALB knockout mice could not produce milk (Stacey et al., 1995; Stinnakre et al., 1994). LALB expression responds differently to lactogenic hormones across species. Prolactin sustains lactose production during lactation, influencing milk composition and osmolality. Milk composition varies among species; for instance, human milk has high lactose (∼70 g/L) (Kim and Yi, 2020), cow’s milk has moderate lactose levels (∼50 g/L) (Heine et al., 2017). However, human milk also has a higher occurrence of milk oligosaccharides (HMOs), containing approximately 12–14 g/L (Kim and Yi, 2020) compared to bovine milk (BMOs) which contains 0.1–1 g/L (Urashima et al., 2013; Chauhan et al., 2023) and lower lactose production may partially explain why this naturally occurs.

Glucose availability in the Golgi is one of the limiting factors of lactose synthesis (Lin et al., 2016), with lactogenic hormones failing to induce glucose transporter (GLUT) expression in bovine mammary cells (Shao et al., 2013) thereby hindering glucose entry into the cell. GLUT1 expression increases in response to oestradiol and progesterone (Medina et al., 2003), redistributing to the Golgi (Lee et al., 2015). GLUT1 upregulation in pregnancy/lactation is linked to serotonin (Laporta et al., 2013) and hypoxia (Chang et al., 2022; Shao et al., 2014). Serotonin also increases GLUT8, while hypoxia and lipopolysaccharide decrease production of this enzyme.

The process of lactose synthesis begins through acquiring glucose and UDP-galactose in the Golgi. Extracellular glucose is taken up by MEC via GLUT1 and sodium-glucose transporter 1 (SGLT1) (Navale and Paranjape, 2016; Riskin et al., 2008; Obermeier et al., 2000). Glucose can either be transported directly into the Golgi apparatus via GLUT1 for lactose synthesis (Nemeth et al., 2000a) or alternatively glucose remaining in the cytoplasm can be phosphorylated by hexokinase (HK) to yield glucose-6-phosphate (G6P) (Sun et al., 2008), which is then subsequently used to create a pool of UDP-bound galactose. GLUT8 was also found in lactating MEC in mice, co-localized with specific Golgi proteins (Villagrán et al., 2019), indicating its involvement in glucose transport during lactose synthesis. SGLT1 was identified in lactating cow MEC (Zhao et al., 1999), but its functional role remains unclear. Studies suggest variable expression of GLUT1, GLUT8, and GLUT12 during pregnancy and lactation in different species (Zhao et al., 1999; Macheda et al., 2003), but their exact contribution to glucose uptake for lactose synthesis in MEC Golgi is not fully understood. Further research is needed to clarify the role of these transporters in lactose synthesis.

**FIGURE 2:**
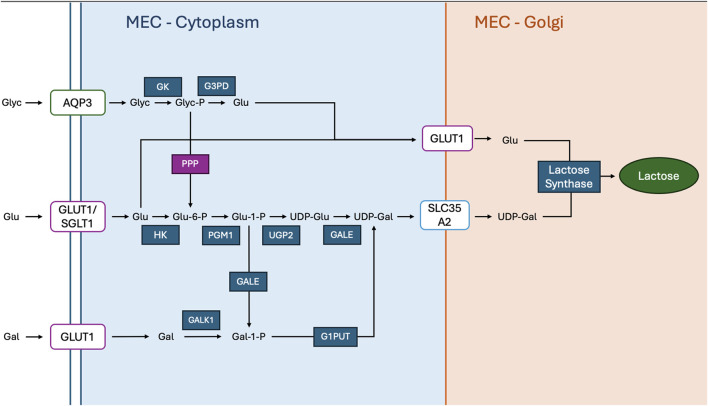
Glucose And UDP-Galactose Are Metabolised In The Golgi Of Mammary Epithelial Cells (MECs) For Lactose Synthesis: Aquaporin 3 (AQP3) facilitates the transport of glycerol (Glyc) across the plasma membrane, where glycerol kinase (GK) phosphorylates it to glycerol-3-phosphate (Glyc-P), and glycerol-3-phosphate dehydrogenase (G3PD) converts it to dihydroxyacetone phosphate (DAP). The pentose phosphate pathway (PPP) branches from glucose metabolism, contributing to nucleotide synthesis and reducing power. Glucose (Glu) is taken up into the cytoplasm via glucose transporter 1 (GLUT1) and sodium-glucose co-transporter 1 (SGLT1), while galactose (Gal) is transported by GLUT1. Inside the cytoplasm, hexokinase (HK) phosphorylates glucose to glucose-6-phosphate (Glu-6-P), which is then converted to glucose-1-phosphate (Glu-1-P) by phosphoglucomutase 1 (PGM1). UDP-glucose pyrophosphorylase 2 (UGP2) synthesizes UDP-glucose (UDP-Glu) from glucose-1-phosphate, and UDP-galactose 4′-epimerase (GALE) converts UDP-glucose to UDP-galactose (UDP-Gal). Simultaneously, galactose is phosphorylated to galactose-1-phosphate (Gal-1-P) by galactokinase 1 (GALK1). galactose-1-phosphate uridylyltransferase (G1PUT) then converts galactose-1-phosphate into UDP-galactose. The solute carrier family 35 member A2 (SLC35A2) transports UDP-galactose into the Golgi apparatus, where glucose and UDP-galactose are combined by lactose synthase to produce lactose.

The *de novo* synthesis of UDP-galactose involves a series of sequential biochemical steps (Figure 2). Initially, phosphoglucomutase (PGM1-3) catalyses the transfer of a phosphate group from the C6 position of glucose-6-phosphate (G6P) (Najjar and Pullman, 1954) to the C1 position of glucose, yielding glucose-1-phosphate (G1P). Following this reaction UDP-glucose-pyrophosphorylase (UGP2) facilitates the exchange of the phosphate group with a UDP moiety, resulting in the formation of UDP-glucose (Duggleby et al., 1996). Subsequently, UDP-glucose is transformed into UDP-galactose via the action of galactose epimerase (GALE) (Schulz et al., 2004), which catalyses the conversion of the glucose moiety to galactose. An alternative pathway involves the conversion of G1P directly to galactose-1-phosphate (Gal1P) through the activity of GALE (Schulz et al., 2004). Following this, galactose-1-phosphate uridylyltransferase (GALT or G1PUT) transfers a UDP moiety to Gal1P, yielding UDP-galactose (Kalckar et al., 1953). UDP-galactose (UDP-Gal) can also be formed from galactose derived from dietary lactose Upon entering the cell, galactose is phosphorylated by the enzyme galactokinase, converting it into galactose-1-phosphate (Gal-1-P) (Holden et al., 2004). This intermediate then reacts with UDP-glucose through the action of the enzyme galactose-1-phosphate uridylyltransferase (GALT or G1PUT), resulting in the formation of UDP-galactose and glucose-1-phosphate (G1P) (Geeganage and Frey, 1999). Once synthesized, UDP-galactose needs to be transported into the Golgi apparatus for further glycosylation reactions. This transportation is facilitated by the UDP-galactose transporter (UGT), encoded by either *SLC35A2 or SLC35B1* genes (Hadley et al., 2019). These transporters play a crucial role in shuttling UDP-galactose across the Golgi membrane, where it serves as a substrate for various glycosyltransferase enzymes involved in glycan biosynthesis.

**FIGURE 3:**
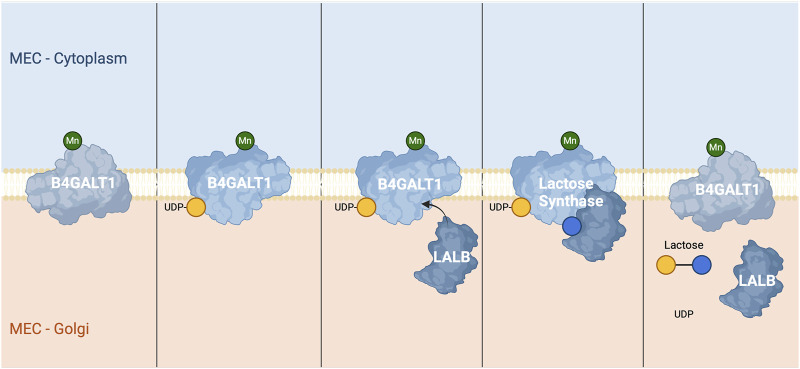
Lactose Synthesis via Lactose Synthase: Lactose synthesis is catalysed by β-1,4-galactosyltransferase 1 (B4GALT1) in the Golgi apparatus of mammary epithelial cells. The process is dependent on manganese (Mn) as a cofactor for enzymatic activity. In the first panel, B4GALT1 is shown embedded in the Golgi membrane. In the second panel, UDP-galactose (UDP-Gal) binds to B4GALT1 in the Golgi lumen. The third panel illustrates the alignment of lactose synthase components, with the protein LALB associating with B4GALT1 to modify its substrate specificity. In the fourth panel, glucose (Glu) binds to the enzyme-substrate complex, positioning itself for the transfer of galactose. The fifth and final panel demonstrates the completion of the reaction, where galactose from UDP-galactose is transferred to glucose, forming lactose. The byproduct UDP is released following the reaction, and lactose is shown as the final product.

The final step of lactose synthesis occurs within the Golgi apparatus, where lactose synthase joins glucose and UDP-galactose by a β-1,4 glycosidic bond. B4GALT1 is responsible for catalysing the final step in lactose synthesis, where it transfers a galactose molecule from UDP-galactose to glucose, forming lactose (Powell and Brew, 1975; Figure 3). UDP-galactose (the donor substrate) typically attaches to B4GALT1 first (Harrus et al., 2018), followed by the binding of glucose (the acceptor substrate) (Powell and Brew, 1975). However, B4GALT1 alone exhibits relatively low affinity and specificity for glucose as a substrate. LALB enables the β1,4-galactosyltransferase to have a high specificity for glucose (Jones, 1972; Neville, 2009), crucial for lactose formation, by inducing a structural change. This is crucial as β1,4-galactosyltransferase in the absence of LALB transfers D-galactose from UDP-galactose to *N*-acetylglucosamine (Qasba et al., 2008).

B4GALT1 undergoes a conformational change upon binding to LALB and glucose (Ramakrishnan et al., 2002). This conformational change is facilitated by the formation of a holoenzyme complex between LALB and B4GALT1 and primarily affects the region spanning residues 345–365, repositioning His347 to coordinate a metal ion and form a sugar-binding site. The Arg359 side chain also reorients, blocking the hydrophobic *N*-acetyl group-binding pocket and facilitating optimal interactions with glucose. These structural adjustments create a conformation that is highly specific for glucose and stabilise it in the acceptor-binding site (Ramakrishnan and Qasba, 2001). Once both glucose and UDP-galactose bind to their individual active site on the lactose synthase enzyme complex, the enzyme then facilitates the transfer of the galactose moiety from UDP-galactose to the C4 hydroxyl (4-OH) group of glucose, forming a β-1,4-glycosidic bond. This bond formation results in the synthesis of lactose, with the release of UDP as a by-product (Sadovnikova et al., 2021). The binding of LALB to B4GALT1 not only alters its substrate specificity but also enhances its catalytic activity, significantly increasing the rate of lactose synthesis.

Lactose then acts as a foundational building block for the biosynthesis of numerous HMOs. Following the initial utilization of lactose, a series of enzymatic processes come into play, directing the transformation of lactose or lactose-derived structures into a diverse array of HMOs. Lactose elongation through the β1,3-linkage leads to linear structures that are para-HMOs. By contrast, the β1,6-linkage introduces chain branching, which leads to the formation of iso-HMOs (Kobata, 2010). These enzymes facilitate intricate modification pathways, orchestrating the conversion of simple sugars into complex oligosaccharides with varying structures and functions.

### 3.2 Fucosylation pathway

Fucosylation involves the addition of fucose residues to precursor molecules, including lactose. Fucose can be added in α1-2, α1-3, or α1-4 linkages (Shan et al., 2019), resulting in different HMO structures. The enzymes responsible for fucose addition are the fucosyltransferases (FUTs), such as FUT1-FUT13, with FUT2,3 and 4 playing the most important role in this mechanism. These enzymes catalyse the transfer of fucose from GDP-fucose as a nucleotide sugar donor to acceptor molecules (Schneider et al., 2017). Fucosylation introduces structural diversity into HMOs, contributing to their biological functions, such as modulation of the gut microbiota and host-microbe interactions.

#### 3.2.1 Fucosyltransferases

The fucosyltransferase family comprises a group of enzymes responsible for catalysing the transfer of fucose residues from a donor substrate, typically GDP-fucose, to acceptor molecules such as oligosaccharides, glycoproteins, glycolipids, and proteins (Wu et al., 2019). Fucose plays essential roles in various biological processes, including cell-cell communication, cell adhesion, immune response, and embryogenesis (Choi et al., 2015). The fucosyltransferase family exhibit diversity in their substrate specificity, subcellular localization, and tissue distribution, reflecting their distinct roles in different biological contexts. Several fucosyltransferases have been identified in mammals, including humans, and are classified into different subtypes based on their substrate specificity and function.

FUT1 and FUT2 are both α1,2-fucosyltransferases. FUT1 is responsible for the formation of the H antigen relating to blood groups (Daniels et al., 2013), which is the precursor for the formation of several other HMOs such as 2′-FL the most predominant HMO. FUT1 catalyses the transfer of a fucose residue from GDP-fucose to the terminal galactose residue of type-2 oligosaccharides in an α1,2 linkage, forming the H-antigen (He and Liu, 2014). FUT2 is closely related to FUT1 and shares similar substrate specificity, both sharing a conserved VGVHVRRGD sequence (Faik et al., 2000). *FUT2* is known as the “Secretor gene” and catalyses the transfer of fucose to the terminal galactose of type-1 oligosaccharides, generating the ABO(H) blood group antigens in mucosal epithelial cells and secretions (He and Liu, 2014) as well as HMOs. Secretor status has important biological implications for host-microbe interactions, immune function, and disease susceptibility. Individuals with an active *FUT2* gene (secretors) produce α1,2-fucosylated HMOs such as 2′-FL, which can shape the infant gut microbiome by promoting beneficial bacteria such as bifidobacteria. In contrast, non-secretors lack these fucosylated HMOs, potentially altering microbial colonization (Van den Abbeele et al., 2019; Zhang et al., 2023) and influencing susceptibility to infections (Hegar et al., 2019), autoimmune diseases, and inflammatory conditions (Fu et al., 2023; Bode, 2018; Abbas et al., 2021).

FUT3, FUT4, FUT5, FUT6 and FUT7 are all α1,3/4-fucosyltransferase enzymes involved in the synthesis of various Lewis blood group antigens (Daniels et al., 2013) with mainly FUT3 and FUT4 being involved in the synthesis of HMOs. Sequence alignment of α1,3/4-fucosyltransferases from a number of different species revealed a highly conserved 17-amino acid sequence, FxL/VxFENS/TxxxxYxTEK, commonly known as the α3-FucT motif (Martin et al., 1997). This conserved region has been suggested to dictate the binding of GDP-fucose, allowing for the catalytic transfer of fucose to its acceptor molecule. *FUT3* is termed the “Lewis gene” and exhibits dual specificity as it can transfer fucose residues to both α1,3 and α1,4 positions of *N*-acetylglucosamine or *N*-acetyllactosamine moieties, giving rise to Le^x^ and Le^a^ respectively. Whereas FUT4 adds fucose only in an α1,3 linkage to *N*-acetyllactosamine. Lewis status influences the structural diversity of HMOs and impacts host-microbe interactions, immune modulation, and susceptibility to infections by shaping the glycan landscape available for recognition by gut bacteria and immune receptors.

The α1,6-fucosyltransferase enzyme, FUT8, catalyses the transfer of fucose to the innermost GlcNAc residue of *N*-glycans attached to glycoproteins (Yang et al., 2021), leading to the formation of core fucosylation. This enzyme is not known to be involved in the HMO synthesis process.

#### 3.2.2 GDP-fucose incorporation to HMOs

**FIGURE 4:**
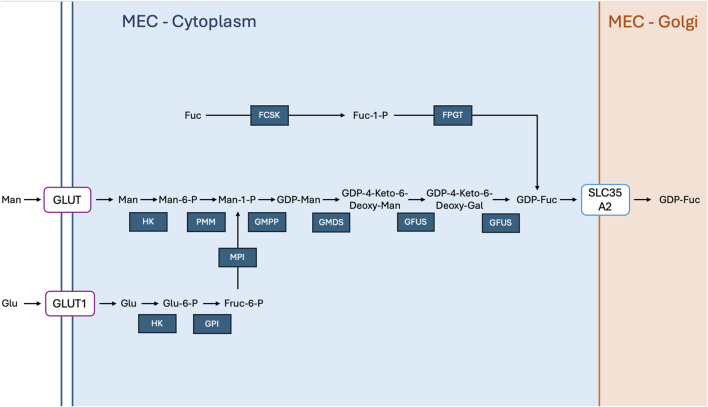
GDP-Fucose Generation In Mammary Epithelial Cells: Free fucose (Fuc) in the cytoplasm is phosphorylated by fucokinase (FCSK) to fucose-1-phosphate (Fuc-1-P), which is then converted to GDP-fucose (GDP-Fuc) by GDP-fucose pyrophosphorylase (FPGT). Mannose (Man) is transported into the cell via glucose transporter (GLUT), where hexokinase (HK) phosphorylates it to mannose-6-phosphate (Man-6-P). Phosphomannomutase (PMM) converts mannose-6-phosphate to mannose-1-phosphate (Man-1-P), which is subsequently transformed into GDP-mannose (GDP-Man) by GDP-mannose pyrophosphorylase (GMPP). GDP-mannose 4,6-dehydratase (GMDS) converts GDP-mannose to GDP-4-keto-6-deoxymannose, which is then converted to GDP-4-keto-6-deoxygalactose by GDP-fucose synthetase (GFUS), and finally to GDP-fucose. Glucose (Glu) enters the cytoplasm via GLUT1 and is phosphorylated by hexokinase (HK) to glucose-6-phosphate (Glu-6-P). Glucose-6-phosphate isomerase (GPI) converts glucose-6-phosphate to fructose-6-phosphate (Fruc-6-P), which is further processed by mannose phosphate isomerase (MPI) to contribute to mannose metabolism. GDP-fucose is transported into the Golgi apparatus by solute carrier family 35 member A2 (SLC35A2), where it participates in glycosylation processes.

GDP-fucose is formed in the cytoplasm through two different enzymatic pathways; the *de novo* pathway which is mannose or glucose dependent (Ginsburg, 1960) or the salvage pathway which utilises free fucose derived from glycoprotein degradation (Park et al., 1998), depending on the organism. In humans, the *de novo* pathway is the primary route for GDP-fucose synthesis (Yurchenco and Atkinson, 1975; Figure 4).

Exogenous fucose or free fucose derived from glycoprotein degradation can also be used to form GDP-fucose. Fucokinase (FCSK) acts on these to give rise to fucose-1-phosphate (F1P) by utilising ATP to catalyse a phosphate group onto the fucose molecule. Next fucose-1-phosphate guanylyltransferase (FPGT) acts upon F1P also ultimately yielding GDP-fucose.

After its synthesis in the cytoplasm, GDP-fucose is transported to the Golgi apparatus (Lübke et al., 1999) where it serves as a substrate for various fucosyltransferases involved in glycoprotein and glycolipid biosynthesis. The transport of GDP-fucose from the cytoplasm to the Golgi involves specialized transporters or translocases located in the membranes of cellular organelles such as SLC35C1, which codes for GDP-Fucose Transporter (Lübke et al., 2001; Yuan and Haltiwanger, 2020). Here, the newly synthesised GDP-fucose can be subjected to the action of numerous different fucosyltransferases, allowing for the incorporation of a fucosyl residue deriving from this molecule, to a chosen acceptor.

Fucosyltransferase has a specific binding pocket for GDP-fucose. The enzyme recognizes the GDP moiety, ensuring that the fucose is correctly positioned for transfer (de Vries et al., 2001). This specificity is crucial for the catalytic activity of FUT. The binding of GDP-fucose to the fucosyltransferase induces conformational changes in the enzyme, positioning the fucose residue optimally for the transfer reaction. For example, binding of GDP-fucose to FUT8 induces substantial structural changes that enable catalysis. Notably, a flexible region comprising residues 428–444 undergoes a large conformational shift to partially enclose the binding site and shield the guanosine portion of GDP-fucose. During this transition, a helical segment (α10) is rearranged, where residues 436–442 unfold, while a new helix (residues 430–435) forms to stabilize the bound substrate. Additionally, another loop (residues 365–378), disordered in the unbound form, becomes structured upon substrate binding. This ordered loop contributes to the formation of both the GDP-fucose binding pocket and the acceptor substrate site, positioning FUT8 in a catalytically active state (García-García et al., 2020). The acceptor molecule in HMO synthesis is typically an oligosaccharide with a free hydroxyl group (often on the terminal sugar unit) that is available for glycosylation. Common acceptors include lactose or other partially synthesized oligosaccharides. The specific type of fucosyltransferase determines the position and orientation of the fucose addition. For example, in the synthesis of 2′-FL, the acceptor molecule is lactose (Galβ1-4Glc) and involves the addition of GDP-L-fucose onto the galactose moiety of lactose by an (α1,2)-fucosyltransferase either FUT1 or FUT2. The fucosyltransferase catalyses the transfer of the fucose moiety from GDP-fucose to the hydroxyl group on the acceptor substrate, forming a glycosidic bond (Iwamori and Domino, 2004; Murray et al., 1997). Other examples are α1,3/4-fucosyltransferases, which add fucose in an α1,3 or α1,4 linkage (Johnson and Watkins, 1992; Murray et al., 1997), leading to the formation of HMOs such as LNFP. After the transfer, GDP is released from the enzyme, which then resets for the next catalytic cycle. The release of GDP is crucial as it prevents product inhibition and allows the enzyme to continue synthesizing fucosylated oligosaccharides efficiently. The specificity of different fucosyltransferases, in terms of both the position of fucose attachment and the choice of acceptor substrate, contributes to the structural diversity of HMOs.

### 3.3 Sialylation pathway

Sialylation involves the addition of sialic acid residues to precursor oligosaccharides. Sialic acid can be added in α2-3, α2-6, or α2-8 linkages to terminal positions of oligosaccharide chains. Various sialyltransferase enzymes catalyse the transfer of sialic acid from CMP-sialic acid, to acceptor molecules (Chen and Varki, 2010; Li and Chen, 2012). Sialylation plays critical roles in host-pathogen interactions, immune modulation, and recognition processes. Sialylated HMOs are involved in the development of the infant immune system and protection against pathogens.

#### 3.3.1 Sialyltransferases

**FIGURE 5:**
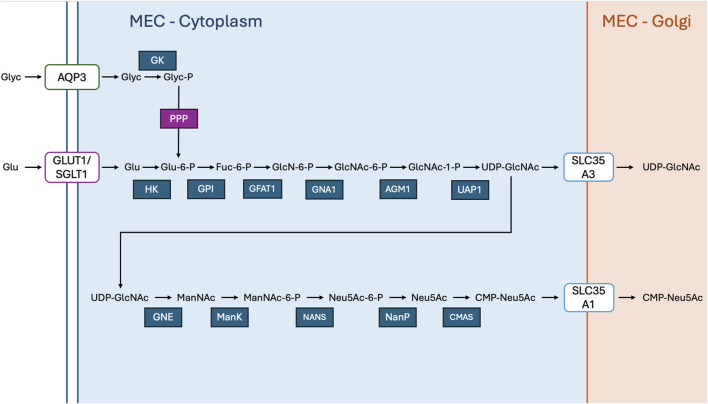
UDP-GlcNAc And CMP-Neu5Ac Synthesis In Mammary Epithelial Cells: In the cytoplasm, glucose (Glu) enters the cell via glucose transporter 1 (GLUT1) and is phosphorylated by hexokinase (HK) to form glucose-6-phosphate (Glu-6-P). This is further metabolized through the hexosamine biosynthetic pathway, converting fructose-6-phosphate (Fuc-6-P) into *N*-acetylglucosamine-6-phosphate (GlcN-6-P) via the enzyme glutamine-fructose-6-phosphate aminotransferase (GFAT). GlcN-6-P is then converted to *N*-acetylglucosamine-1-phosphate (GlcNAc-1-P) by phosphoglucosamine mutase (PGM3), followed by uridine diphosphate (UDP) activation through UDP*-N-*acetylglucosamine pyrophosphorylase (UAP1) to form UDP-GlcNAc. This nucleotide sugar is then transported into the Golgi via solute carrier family 35 member A2 (SLC35A2) for glycosylation reactions. Simultaneously, UDP-GlcNAc serves as a precursor for sialic acid biosynthesis. *N*-acetylglucosamine (GlcNAc) is converted to *N*-Acetylmannosamine (ManNAc) by glucosamine-6-phosphate *N*-acetyltransferase (GNE), which is further phosphorylated by *N*-Acetylmannosamine kinase (NANK) to form *N*-acetylmannosamine-6-phosphate (ManNAc-6-P). This is then converted into *N*-acetylneuraminic acid (Neu5Ac) via *N*-acetylneuraminate synthase (NANS). Neu5Ac is subsequently activated to CMP-Neu5Ac by CMP*-N-*acetylneuraminic acid synthetase (CMAS). The activated CMP-Neu5Ac is transported into the Golgi by solute carrier family 35 member A1 (SLC35A1) for incorporation into glycoproteins and glycolipids.

The sialyltransferase family comprises a group of enzymes responsible for catalysing the transfer of sialic acid residues from activated donor substrates, typically CMP-sialic acid or UDP-sialic acid, to acceptor molecules (Figure 5; Li and Chen, 2012). Sialyltransferases play crucial roles in the biosynthesis of sialylated glycoconjugates, including glycoproteins, glycolipids, and proteoglycans (Harduin-Lepers et al., 2005), which are involved in various biological processes such as cell-cell recognition, immune response, and pathogen interactions. Sialyltransferases are traditionally grouped into four families based on the type of glycosidic linkage they form (α2,3-, α2,6-, or α2,8-) and their main monosaccharide acceptor, such as galactose *N*-acetylgalactosamine, or another sialic acid residue. These families are named ST3Gal, ST6Gal, ST6GalNAc, and ST8Sia (Petit et al., 2015) and unlike other glycosyltransferases, contain two conserved protein domains known as ‘sialylmotifs,’ found in all cloned sialyltransferases. The larger ‘L-sialylmotif’ (48–49 amino acids) resides in the *N-*terminal catalytic domain and includes eight invariant residues, while the smaller ‘S-sialylmotif’ (23 amino acids) near the C-terminal contains two invariant residues, with limited variability in other residues. Site-directed mutagenesis of ST6Gal I showed that the ‘L-sialylmotif’ binds the donor substrate CMP-Neu5Ac, and the ‘S-sialylmotif’ aids binding of both donor and acceptor substrates (Datta and Paulson, 1997). In the context of HMO synthesis, sialyltransferases are responsible for the transfer of sialic acid residues such as *N*-acetylneuraminic acid (NeuAc or Neu5Ac) to the HMO chain. *N*-glycolylneuraminic acid (NeuGc) is a sialic acid that exists in some mammalian tissues but is not present in human milk. There are several sialyltransferases involved in HMO biosynthesis, including ST3GAL1, ST6GAL1, and ST6GALNAC2 (Li and Chen, 2012). These enzymes catalyse the formation of several sialylated HMOs, including 3′-SL and 6′-SL.

The ST3 β-galactoside α-2,3-sialyltransferase (ST3GAL) subtype of sialyltransferase catalyses the transfer of sialic acid residues in an α-2,3 linkage to galactose residues on glycoproteins and glycolipids and contains 6 family members (ST3GAL1-6) (Petit et al., 2015). ST3GAL1 as well as ST3GAL2 show high activity toward Galβ1,3GalNAc (type III) oligosaccharides and minimal activity to Galβ1,4GlcNAc (type II) (Kono et al., 1997; Gupta et al., 2016), allowing them to transfer Neu5Ac to the galactose residues of these oligosaccharides. Deletion studies in COS-7 cells revealed that *N-*terminal regions are critical for enzymatic activity and secretion, with active soluble forms, hST3-Δ25 (with the first 25 amino acids of the sequence deleted) and hST3-Δ56 (with the first 25 amino acids of the sequence deleted) being glycosylated at all five *N*-glycosylation sites of these enzymes (Vallejo-Ruiz et al., 2001). Further mutational studies on ST3GAL1 revealed specific residues (His299, Tyr300, and His316) are essential for activity, while *N*-glycosylation, though not required for enzymatic function, supports proper folding and trafficking of the enzyme (Jeanneau et al., 2004). ST3GAL3 and ST3GAL4 exhibit high activity toward the type I and II disaccharides, but opposite to their ST3GAL1 and 2 family members have very low activity toward type III disaccharides (Kono et al., 1997). ST3GAL5 is a sialyltransferase that is recognised for catalysing the addition of α2,3 sialic acids to glycosphingolipids and is linked to various cancers if mutated (Uemura et al., 2014; van der Haar Àvila et al., 2024). ST3GAL6 also shows enzymatic activity toward the Galβ1,4GlcNAc structure on glycoproteins and glycolipids, exhibiting restricted substrate specificity (Okajima et al., 1999).

ST6 β-galactoside α-2,6-sialyltransferase (ST6GAL) catalyses the transfer of sialic acid residues in an α-2,6 linkage to galactose residues on glycoproteins and glycolipids. This subtype of sialyltransferase is involved in the synthesis of sialylated glycoconjugates found on cell surface receptors and plays crucial roles in cell signaling, cell adhesion, and immune regulation (Hennet et al., 1998a). ST6GAL2 is a variant of ST6GAL that exhibits a similar substrate specificity but may have distinct tissue expression patterns and biological functions. It is involved in the synthesis of α-2,6-sialylated glycoconjugates and contributes to cell surface glycan diversity and function. The role of *N*-linked glycosylation in ST6GAL1 was evaluated, revealing that glycosylation at Asn146 and Asn158 is not essential for ER-to-Golgi transport of the membrane-associated form but contributes to protein stability. Soluble forms of this enzyme require glycosylation for secretion and activity, while unglycosylated mutants remain ER-retained and inactive, highlighting distinct glycosylation needs for membrane-bound versus soluble forms (Chen and Colley, 2000).

ST6 α-*N*-acetyl-galactosaminyl α-2,6-sialyltransferase (ST6GALNAC) catalyses the transfer of sialic acid residues in an α-2,6 linkage to the 6-hydroxyl group of *N*-acetylgalactosamine residues on glycoproteins (Harduin-Lepers et al., 2001). ST6GALNAC family contains six family members (Li et al., 2023) and can be broadly grouped into two subfamilies. ST6GALNAC1, and 2 specifically form α2,6 linkages on GalNAc residues within *O*-glycans (Pei et al., 2024; Lee et al., 1999), with ST6GALNAC1 exhibiting a preference for GalNAc-Ser/Thr, while ST6GALNAC2 primarily modifies Galβ1,3GalNAc-Ser/Thr (Okajima et al., 2000). ST6GALNAC3, 4, 5, and 6 target GalNAc residues in gangliosides for sialylation (Pei et al., 2024) and also possess a similar substrate specificity, i.e., a terminal sialic acid with an α2,3 linkage on galactose was essential as an acceptor structure. This family has also been suggested to be responsible for α-2,6-sialylation of GlcNAc giving rise to HMOs such as LSTb and DSLNT as humans do not have a dedicated ST6GlcNAc enzyme to carry out this activity, with ST6GALNAC 3, 5 and 6 all being plausible candidates (Bao et al., 2024; Prudden et al., 2017).

#### 3.3.2 CMP-Neu5Ac incorporation to HMOs

The synthesis of CMP-Neu5Ac involves several enzymatic steps within the cytoplasmic compartment of cells. SLC35A1 is the specific nucleotide sugar transporter responsible for transporting CMP-Neu5Ac from the cytosol into the Golgi lumen (Moskovskich et al., 2019). The synthesized CMP-Neu5Ac is then utilized as a substrate by various sialyltransferases within the Golgi apparatus for the sialylation of glycoproteins and glycolipids, contributing to HMO structural and functional diversity. Much like fucosyltransferases, sialyltransferases also have a highly specific binding site for their activated sugar, in this case CMP-Neu5Ac, which ensures that only sialic acid in its activated form is transferred (Schelch et al., 2020). The CMP moiety of CMP-Neu5Ac interacts with the enzyme, positioning Neu5Ac for optimal transfer. Binding of CMP-Neu5Ac to the enzyme induces a conformational change, which is critical for catalytic activity (Hugonnet et al., 2021). The crystal structure of HsST6GAL1 revealed that binding of CMP-Neu5Ac induces a change that stabilises the catalytic loop and repositions key sialylmotifs for catalysis (Datta et al., 2001). Tyr354 interacts with both the phosphate and sialic acid moieties even in the apo form, suggesting pre-formed donor recognition. Upon ligand binding, residues 366–372 (motif “d” and sialylmotif VS) become ordered, the disulfide bond Cys353–Cys364 shifts orientation, and the C-5 side chain of Neu5Ac aligns toward solvent-exposed space, enabling specific motif interactions. This change aligns the sialic acid moiety with the acceptor substrate for the glycosylation reaction.

The acceptor substrate for sialyltransferases in HMO synthesis is usually an oligosaccharide that contains a free hydroxyl group on a terminal sugar residue, such as galactose (Gal) or *N*-acetylglucosamine (GlcNAc). In the formation of 3′-SL, the acceptor molecule is lactose. The sialyltransferase catalyses the transfer of the Neu5Ac moiety from CMP-Neu5Ac to the acceptor substrate, forming a sialylated glycosidic bond (Zhu Y. et al., 2023). The position and linkage of the sialic acid residue depend on the specific sialyltransferase. For example, α2,3-sialyltransferases add Neu5Ac in an α2,3 linkage to the galactose of lactose, forming 3′-SL. On the other hand, α2,6-sialyltransferases add Neu5Ac in an α2,6 linkage (Zhou et al., 2023), which is less common in HMOs but found in other glycoconjugates. Sialyltransferases exhibit high specificity for both the donor (CMP-Neu5Ac) and the acceptor substrate, determining the specific structure and biological function of the resulting sialylated HMOs.

### 3.4 Elaboration pathways

Glycosyltransferase enzymes can also sequentially add monosaccharide residues to precursor oligosaccharides, leading to the formation of structurally diverse HMOs. The glycosyltransferases involved in the elaboration of oligosaccharides include β1-4 galactosyltransferase, α1-2 fucosyltransferase, α1-3/4 fucosyltransferase, and sialyltransferases. The sequential addition of monosaccharides results in the generation of a wide range of HMO structures with varying glycosidic linkages and branching patterns, contributing to the complexity and functionality of human milk. Further modification of HMOs increases their structural diversity and may enhance their biological activities, such as anti-adhesive properties against pathogens.

#### 3.4.1 Galactosyltransferases

The galactosyltransferase family comprises a group of enzymes responsible for catalysing the transfer of galactose residues from activated donor substrates, such as UDP-galactose or GDP-galactose, to acceptor molecules (Qasba et al., 2008). These enzymes play essential roles in the biosynthesis of glycoconjugates, including glycoproteins, glycolipids, and proteoglycans, which are crucial for various biological processes such as cell-cell recognition, cell adhesion, and signaling. Members of the galactosyltransferase family share a conserved glycosyltransferase domain and are classified into different subtypes based on their substrate specificity, acceptor molecule, and cellular localization. Subfamilies of galactosyltransferases include the inverting glycosyltransferases β1-4 (β4GalT), β1-3 (β3GalT), and β1-6 (β6GalT), and retaining galactosyltransferases, α1-3 (α3GalT) and α1-4 (α4GalT), which generate β1-4, β1-3, β1-6, α1-3, and α1-4 linkages, respectively (Hennet, 2002). There are several galactosyltransferases involved in HMO biosynthesis, including B3GALT2 and B4GALT1 (Qasba et al., 2008) with the B3GALT and B4GALT families being the most recognised as being involved in the HMO formation process. These enzymes catalyse the formation of several galactosylated HMOs, including lacto*-N-*tetraose and lacto*-N-*neotetraose.

β-1,3-Galactosyltransferases (B3GALT) transfer galactose residues from UDP-galactose to acceptor molecules, forming β-1,3-glycosidic linkages. This gene family contains homologous genes encoding for these type-II membrane proteins (Amado et al., 1999; Petit et al., 2021). These enzymes are involved in the synthesis of specific glycosaminoglycans (GAGs) and proteoglycans, contributing to the structural integrity of extracellular matrices and cellular signaling. There is little homology between B3GALT and B4GALT proteins, indicating distinct evolutionary origins (Hennet, 2002). In the context of human milk oligosaccharide synthesis, several B3GALT family members, including B3GALT1 and B3GALT2, are particularly important. These enzymes contribute to forming the galactose-containing core structures of HMOs (Hennet et al., 1998b), facilitating the production of biologically active oligosaccharides. B3GALT1 is well-known for its role in the biosynthesis of lacto*-N-*biose, a key precursor in HMO synthesis, and other β-1,3-galactosylated structures (Trinchera et al., 2014). Meanwhile, B3GALT4 is involved in the formation of gangliosides, which are crucial components of neural cell membranes. B3GALT5 plays a critical role in the biosynthesis and regulation of type 1 chain oligosaccharides, particularly in cancer-associated glycosylation patterns. Elevated B3GALT5 expression correlates with increased synthesis of type 1 chain derivatives such as Lewis a (Le^a^) and sialyl-Lewis a (sLe^a^) (Mare and Trinchera, 2004; Lin et al., 2009). B3GALT6 transfers galactose from UDP-galactose to substrates with a terminal β-linked galactose residue and is most well-known for its role in GAG biosynthesis (Bai et al., 2001), specifically the GAG linker region (Nakajima et al., 2013).

β-1,4-Galactosyltransferases (B4GALT) are a subfamily of galactosyltransferases which contains seven different family members (B4GALT1-7), all but B4GALT7 sharing four conserved catalytic domain located cysteine residues (Amado et al., 1999). These enzymes catalyse the transfer of galactose from UDP-galactose to acceptor molecules, typically forming β-1,4-glycosidic linkages. B4GALTs are involved in the biosynthesis of various glycoconjugates, including glycoproteins and glycolipids, and play important roles in cell surface carbohydrate structures. B4GALT1 was the first mammalian galactosyltransferase to be characterised (Hennet, 2002). This enzyme is one of the primary units that makes up lactose synthase, responsible for the formation of lactose from glucose and galactose (Ramakrishnan and Qasba, 2001; Brodbeck et al., 1967). Additionally in its singular form, B4GALT1 can catalyse galactose to GlcNAc acceptors. B4GALT2 has been shown to be the closest related family member to B4GALT1, structurally and functionally, closely followed by B4GALT3 (Almeida et al., 1997). B4GALT2 and B4GALT3 are responsible for the synthesis of complex-type *N*-linked oligosaccharides in many glycoproteins as well as the carbohydrate moieties of glycolipids (Almeida et al., 1997). B4GALT4 is recognised for its ability to transfer a gal residue via a β-1,4 linkage and this galactosyltransferase is involved in the synthesis of terminal *N*-acetyllactosamine (LacNac) unit present on glycan chains of glycoproteins (Shauchuk et al., 2020). It was also shown to serve as a major control point for glycan branching in *N*-linked glycosylation, highlighting its critical regulatory role in glycan structural diversity (McDonald et al., 2014). B4GALT5, 6 and 7 are not involved in the HMO biosynthesis process but, B4GALT5 and B4GALT6 are recognised to catalyse the synthesis of lactosylceramide (LacCer) via the transfer of galactose from UDP-galactose to glucosylceramide (GlcCer) (Yamaji and Hanada, 2014) and B4GALT7 is thought to be required for the biosynthesis of the tetrasaccharide linkage region of proteoglycans (Tsutsui et al., 2013).

#### 3.4.2 *N*-acetylglucosaminyltransferase

The *N*-acetylglucosaminyltransferase family comprises a group of enzymes responsible for catalysing the transfer of *N*-acetylglucosamine (GlcNAc) residues from an activated donor substrate, typically UDP-*N*-acetylglucosamine (UDP-GlcNAc), to acceptor molecules, in this case to the HMO chain. There are several GlcNAc-transferases involved in HMO biosynthesis, including B3GNT2 (Janetzko and Walker, 2014). These enzymes catalyse the formation of numerous linear HMOs, including lacto-*N*-tetraose (LNT) and lacto-*N*-neotetraose (LNnT).

β1,3-*N*-acetylglucosaminyltransferases (B3GNTs) are a family of glycosyltransferases crucial for synthesizing poly-*N*-acetyl-lactosamine chains. Their primary function is to catalyse the attachment of *N*-acetylglucosamine to *N*-acetyl-lactosamine repeats (Shiraishi et al., 2001), a vital step in the elongation of these chains. There are currently seven members of this glycosyltransferase subfamily that have been identified (B3GNT2-8) (Hao et al., 2021). B3GNT2 is most widely recognised as the most active member of this family (Togayachi et al., 2006), being primarily responsible for the transfer of GlcNAc from UDP-GlcNAc to an acceptor with a terminal galactose residue (Prudden et al., 2017). B3GNT3 catalyses the extension of core 1 oligosaccharides and contributes to the backbone structure of dimeric sialyl Lewis A (Shiraishi et al., 2001; Yeh et al., 2001). The other B3GNT enzymes (B3GNT4, B3GNT5, B3GNT6, B3GNT7, and B3GNT8) have been more closely linked to the synthesis of glycosaminoglycans and glycosphingolipids (Henion et al., 2001), rather than direct involvement in HMO biosynthesis.

The GlcNAc transferase family also includes the β-1,6-*N*-acetylglucosaminyltransferases, commonly known as GCNTs. These enzymes catalyse the transfer of *N*-acetylglucosamine (GlcNAc) residues from UDP-GlcNAc to the core mannose residues of *N*-linked glycans, specifically adding GlcNAc in a β-1,6 linkage. There are four members of the GCNT family, each with distinct substrate specificities and tissue distributions. GCNT1 is primarily involved in the synthesis of core 2 *O*-glycans (Bierhuizen and Fukuda, 1992), which are commonly found on mucin-type glycoproteins. This enzyme adds GlcNAc in a β-1,6 linkage to the core GalNAc residue, resulting in the formation of the core 2 structure (Ali et al., 2012). By forming core-2 type structures, GCNT1 could potentially contribute to the branching complexity of HMOs, as *in silico* studies have identified GCNT1 as the enzyme responsible for the branching β-1,6-GlcNAc addition (Kellman et al., 2022). This branching is crucial for the functional diversity of HMOs, influencing their biological activities. GCNT2 is involved in the synthesis of core 2 *O*-glycans similar to GCNT1. GCNT2, is responsible for the creation of branched HMOs, by adding a β1,6-linked GlcNAc to an internal galactosyl unit. This branching process can be repeated, with the newly formed β6 arm undergoing further galactosylation, branch extension, and eventually selective fucosylation or sialylation. As a result, complex and asymmetric triantennary HMOs are produced (Prudden et al., 2017). GCNT3 is involved in the synthesis of core 3 *O*-glycans. This enzyme adds GlcNAc in a β-1.6 linkage to the core galactose residue, resulting in the formation of the core 3 structure (Kellman et al., 2022). GCNT4 is another member of the GCNT family involved in *O*-glycan biosynthesis. It has been implicated in the biosynthesis of branched *O*-glycans (Schwientek et al., 2000), although its exact substrate specificity and biological functions are still being elucidated.

#### 3.4.3 UDP-gal and UDP-GlcNAc incorporation to HMOs

UDP-Gal is critical in the biosynthesis of HMOs, acting as a galactose donor in glycosylation reactions (Hayes and Varki, 1992). This process is driven by glycosyltransferase enzymes, which transfer galactose from UDP-Gal to acceptor molecules, typically starting with a lactose core. The enzyme β-1,4-galactosyltransferase, for example, adds a galactose residue to the growing HMO chain (Qasba et al., 2008). As the synthesis progresses, additional galactose residues are incorporated, leading to the elongation and branching of HMOs. These complex structures are further diversified by the addition of other sugars such as *N*-acetylglucosamine, with the incorporation of galactose from UDP-Gal being a key step in generating the variety of HMOs present in human milk.

The biosynthesis of UDP*-N-*acetylglucosamine (UDP-GlcNAc) also begins with glucose (Figure 5). UDP*-N-*acetylglucosamine (UDP-GlcNAc) provides *N*-acetylglucosamine (GlcNAc) for glycosylation (Li et al., 2021). Glycosyltransferases facilitate the attachment of GlcNAc from UDP-GlcNAc to developing HMO structures, typically linked in β-1.3 or β-1.6 configurations, which extends the HMO chains and contributes to the complexity of the oligosaccharides (Sasai et al., 2002). This process is integral to generating the variety of HMOs, with the addition of GlcNAc playing a significant role in shaping their diverse structures.

## 4 Current industrial methods of human milk oligosaccharide synthesis, separation and detection

The current industrial approaches to HMO synthesis primarily involve microbial fermentation with genetically engineered bacteria or yeast, enzymatic synthesis through glycosyltransferases and glycosidases, chemical synthesis, and chemoenzymatic methods. The current synthesis methods used to create HMOs have been widely reviewed (Pérez-Escalante et al., 2022; Zhu et al., 2023b; Gan et al., 2023; Xu and Townsend, 2021) focusing on optimising production for use in infant formula and other health-related products. Glycosyltransferases play a critical role in these methods, driving the precision and diversity of HMO biosynthesis. While the repertoire of glycosyltransferases involved in HMO biosynthesis is well characterised, efficient reconstruction of these pathways for synthetic or industrial purposes remains a significant challenge due to enzymatic limitations and bottlenecks. Several studies have highlighted that not all glycosyltransferases involved in HMO biosynthesis are equally productive (Kellman et al., 2022); some exhibit lower catalytic efficiencies or poor expression profiles in heterologous systems, posing hurdles for scalable production. In the case of lactose biosynthesis, although the enzymatic complex lactose synthase (composed of B4GALT1 and α-lactalbumin) is functionally characterised, the rate-limiting step is not always the enzymatic activity itself but rather the availability of glucose in the Golgi apparatus (Nemeth et al., 2000b), where UDP-galactose is preferentially utilised. In milk oligosaccharide production, the rate-limiting steps often involve the enzymes responsible for fucosylation and other glycosylation reactions, especially in the final steps of biosynthesis, such as the addition of fucose to galactose or N-acetylglucosamine. In engineered systems, this has necessitated innovations to increase intracellular glucose trafficking or utilisation efficiency. However, because these are enzymes from higher eukaryotes, it was quite difficult to express glycosyltransferases recombinantly in *E. coli.* In recent years, it has been determined that bacterial cell surface glycans also have oligosaccharide structures similar to those of mammalian glycans, which indicates that bacteria also serve as a source of glycosyltransferases and can be expressed in *E. coli*. Recent attempts to mimic these pathways have employed microbial hosts such as *E. coli* and *Saccharomyces cerevisiae* (Liu et al., 2025), taking advantage of their ability to express bacterial homologs of glycosyltransferases, which are often more tractable and soluble than their mammalian counterparts. Moreover, directed evolution, rational mutagenesis, and structure-guided engineering have been applied to enhance substrate affinity, broaden donor/acceptor specificity, and improve stability of key GTs involved in HMO synthesis (Liu et al., 2025). These approaches have led to significant advances in one-pot multienzyme (OPME) systems and *in vivo* HMO biosynthesis platforms (Zeuner et al., 2019), although further optimisation is needed to overcome current limitations in yield and structural diversity.

Understanding and addressing these enzymatic bottlenecks is essential not only for improving HMO production but also for expanding the library of accessible glycan structures for functional studies and therapeutic development. Advancing HMO production involves overcoming challenges in scaling glycosyltransferase activity through improved isolation techniques, enhanced enzyme stability, and efficient expression in microbial or mammalian cell cultures. These efforts aim to replicate the benefits of natural HMOs, making them accessible for commercial and therapeutic applications to support infant health and development.

### 4.1 Chemical synthesis

Chemical synthesis involves assembling complex carbohydrate molecules through a series of carefully orchestrated chemical reactions (Ohtsuka et al., 1982). In the context of HMOs, the core of this process is the glycosylation reaction, where glycosidic bonds between sugar units are formed by reacting an activated glycosyl donor, modified to be highly reactive, with a glycosyl acceptor that has a free hydroxyl group. This reaction is driven by activating agents or catalysts that facilitate the precise formation of these bonds (Lv et al., 2023). Chemical synthesis provides extensive control over the final HMO product, allowing for manipulation of its structure, bond configuration (α or β), regioselectivity (the position of glycosidic bonds), chain length, and branching patterns.

The chemical synthesis of HMOs has been extensively reviewed (Zhu et al., 2023c), with several studies outlining advancements in optimized synthetic routes for complex oligosaccharides. Existing reviews highlight the challenges associated with regio- and stereoselectivity in HMO synthesis and discuss the scalability of chemical approaches for industrial applications (Niharika and Singh, 2025). A recent example of successful HMO synthesis is the total chemical synthesis of para-Lacto-*N*-Hexaose (pLNH), para-Lacto-*N*-neohexaose (pLNnH) (Singh et al., 2023), Lacto-*N*-neohexaose (LNnH) (Bandara et al., 2020), Lacto-*N*-neotetraose (LNnT) (Bandara et al., 2019), demonstrating efficient methodologies for assembling these structures with high purity and yield. The commercial interest in chemically synthesized HMOs is evident by the presence of many patents filed by biotechnology and food companies, aiming to refine synthetic methods and enhance HMO chemical synthesis. Patent CN116217633A, for example, covers the synthesis method of double-branched HMOs. As a result of these advancements, chemically synthesized HMOs such as 2′-FL and lacto-*N*-tetraose (LNT) are now available on the market (EFSA Panel on Dietetic Products N, 2015), incorporated into infant formula and functional food products to mimic the composition of human milk and support infant health.

While this method allows for the creation of a diverse array of HMO structures with high precision, it is often complex, time-consuming, and expensive. The process typically involves multiple steps, each requiring careful protection and deprotection of functional groups to ensure the correct bonds are formed, often under harsh reaction conditions that can affect yield and purity. Despite these challenges, chemical synthesis is invaluable for producing specific HMOs that may not be easily obtained through biological methods, offering the ability to create highly defined oligosaccharide structures.

### 4.2 Enzymatic synthesis

Enzymatic synthesis of HMOs leverages the precision of specific enzymes, to catalyse the assembly of HMOs from simpler sugar molecules. This approach closely mirrors the natural biosynthetic pathways that occur in the human body, where enzymes guide the formation of complex carbohydrate structures with exacting accuracy. Enzymatic synthesis stands out for HMO synthesis due to its high selectivity and mild reaction conditions, making it a sustainable and efficient method for producing HMOs. Several reviews provide in-depth discussions on the catalytic mechanisms of glycosyltransferases relating to the optimization of enzyme-based HMO synthesis for industrial applications (Tseng et al., 2024; Zheng et al., 2022a).

A variety of enzymes, including glycosyltransferases and glycosidases, are employed in these pathways to facilitate the precise construction of oligosaccharide chains. Glycosyltransferases catalyse the transfer of sugar moieties from donor molecules to acceptor substrates, forming glycosidic bonds (Chang et al., 2011), while glycosidases hydrolyse glycosidic bonds, breaking down oligosaccharides into simpler sugars (Katayama et al., 2004). This enzymatic toolkit enables the production of a wide range of HMO structures that closely resemble those naturally present in human milk. The high specificity of enzymatic methods ensures that the resulting HMOs have the correct regioselectivity, stereochemistry, and branching patterns, making this approach particularly suited for creating complex and bioactive HMO molecules.

Despite its advantages, enzymatic synthesis has limitations. The inherent selectivity of enzymes means that while they can efficiently produce specific HMOs, there is less flexibility to alter the structure compared to chemical synthesis. This is because the enzymes dictate the final product, restricting the range of possible modifications. Moreover, although enzymes are highly effective in generating complex HMO structures, the practical application of this method is often limited by the challenges of obtaining these enzymes in sufficient quantities and at a reasonable cost. Moremen and colleagues addressed the challenges associated with the recombinant expression of human glycosylation enzymes, which are pivotal in the biosynthesis of vertebrate glycoproteins and glycolipids. These enzymes, including glycosyltransferases, glycoside hydrolases, and sulfotransferases, are known for their specificity, yet their structural and functional analyses have been limited due to difficulties in producing them as functional recombinant proteins. The researchers developed a modular expression vector library encompassing all known human glycosylation-related enzymes (Moremen et al., 2018). The modular expression system enables flexible assembly of expression constructs by combining secretion signals, purification tags, and catalytic domains, which facilitates efficient production and screening of glycosylation enzymes for structural and functional studies. The group expressed these enzymes as secreted catalytic domain fusion proteins in both mammalian and insect cell systems. A subset of these enzymes was successfully purified and characterized, and the crystal structure of the sialyltransferase ST6GALNAC2 was determined. While many enzymes were produced at high yields in both expression systems, individual expression levels varied. This comprehensive expression vector library is anticipated to be a transformative resource, as it provides a standardised, scalable platform for the efficient production of all known human glycosylation enzymes, facilitating recombinant enzyme production and enabling extensive structure–function studies, thereby advancing applications in glycobiology.

Currently, enzymatic synthesis has successfully produced a limited number of HMOs, mainly focusing on relatively simple structures such as 6′-SL (Guo et al., 2018). Patent WO2025015168A1 details a method for producing fucosylated oligosaccharides, LNFP I-III using engineered fucosyltransferase enzymes FUT1 and FUT4. The patent emphasizes the optimization of enzyme activity and stability to improve yield and scalability in industrial applications. Patent CN119040414A details an enzymatic method of producing DSLNT. Despite advances in characterizing mammalian enzymes and purifying glycosyltransferases, scaling up these processes for broader HMO production remains a significant challenge. The economic feasibility and availability of these enzymes are key hurdles that need to be addressed for more widespread application of enzymatic synthesis in the production of HMOs.

### 4.3 Chemoenzymatic synthesis

Chemoenzymatic synthesis integrates the strengths of both chemical and enzymatic methods to produce HMOs with high efficiency and precision. In this hybrid approach, chemical synthesis is initially used to generate key precursors or building blocks, which are then enzymatically assembled into the final oligosaccharide structures (Zheng et al., 2022a). This combination leverages the robustness and versatility of chemical methods with the high specificity and mild conditions of enzymatic reactions. The resulting process enhances the efficiency and selectivity of HMO production, enabling the creation of diverse and structurally accurate HMOs. By addressing some of the limitations inherent in purely chemical or enzymatic methods, chemoenzymatic synthesis offers a more sustainable and effective route to producing complex carbohydrate structures. Reviews on this topic have examined the benefits of integrating chemical and enzymatic approaches to improve both yield and structural diversity while maintaining cost-effectiveness (Bode et al., 2016; Zheng et al., 2022b). Recent studies reported the synthesis of branched HMOs using a versatile chemoenzymatic strategy, enabling the production of diverse HMO structures (Tseng et al., 2025a; Ooi et al., 2022; Tseng et al., 2025b). Tseng et al. exemplifies a chemoenzymatic approach to HMO synthesis by leveraging the substrate promiscuity of human GCNT2 and bacterial glycosyltransferases (GTs) to construct a universal tetrasaccharide core. Using *N*-trifluoroacetyl glucosamine (GlcNTFA) as a chemical intermediate, glycan elongation was controlled through selective chemical modifications, enabling the synthesis of diverse, multiantennary HMOs (Tseng et al., 2025b), Patents CN118325989A and CN118166049A disclose methods for synthesizing asymmetrical branched LNH and LNnH respectively, utilizing a combination of chemical and enzymatic steps to achieve the desired structure.

### 4.4 Microbial fermentation synthesis

Microbial fermentation for the synthesis of HMOs utilises genetically engineered microorganisms, such as bacteria or yeast, to produce HMOs in significant quantities (Endo et al., 2000; Drouillard et al., 2010; Liu et al., 2018). Advances in strain engineering, fermentation optimization, and metabolic pathway modifications to enhance HMO yields are reviewed elsewhere (Bode et al., 2016; Palur et al., 2023). These microorganisms are specifically modified to express the enzymes required for HMO biosynthesis including glycosyltransferases, enabling them to efficiently assemble these complex carbohydrates from precursors provided in the fermentation medium. The engineered microbes are cultivated in large-scale fermentation tanks under carefully controlled conditions, including temperature, pH, and nutrient availability, to optimize growth and productivity. During the fermentation process, the microbes metabolize simple sugars or other carbon sources, using them as substrates to drive the enzymatic assembly of HMO structures (Palur et al., 2023). As they grow and metabolize these substrates, the introduced enzymes catalyse the step-by-step formation of specific HMOs, which are then secreted into the surrounding fermentation broth. Following fermentation, the HMOs are extracted from the broth and undergo a purification process to remove microbial cells, proteins, and other impurities, resulting in the production of pure, desired oligosaccharides.

This method harnesses the natural biosynthetic capabilities and efficiency of microbes, making it a highly promising approach for the large-scale production of HMOs. It offers scalability and potential cost-effectiveness, aligning well with sustainable production practices by utilizing renewable resources and operating under mild, environmentally friendly conditions. However, successful implementation may require careful optimization of culture conditions and advanced genetic engineering techniques to maximize HMO yield and quality. Metabolic engineering of microbial hosts, such as *E. coli* and *S. cerevisiae*, could enhance HMO production. By optimizing the expression of glycosyltransferases and the availability of nucleotide sugar donors, metabolic pathways can be reconfigured to increase HMO yield and diversity. Enzymatic engineering, including directed evolution and rational design, can improve the catalytic efficiency, substrate specificity, and stability of glycosyltransferases. Techniques such as site-directed mutagenesis and high-throughput screening enable the identification of beneficial mutations, leading to more robust enzymes for HMO synthesis. Optimizing the conditions for enzymatic reactions, including pH, temperature, and substrate concentrations, is crucial for maximizing HMO yield and purity. To address scalability and cost-efficiency in microbial fermentation for HMO production, optimizing key factors such as improving microbial strains for higher yields, enhancing precursor availability, refining enzyme expression systems, and minimizing byproduct formation will help. Such measures will allow for a self-sufficient and more sustainable production process, reducing dependency on expensive or limited natural sources for HMO production. Additionally engineering hosts such as *E. coli* to produce multiple HMOs simultaneously could enable the scalable production of complex HMO mixtures that more closely mimic the composition of human milk. Continuous monitoring and adjustment of reaction parameters can help achieve optimal production conditions. Another hurdle in microbial HMO production is the risk of endotoxin contamination, particularly when using Gram-negative hosts such as *E*. *coli*, necessitating additional purification steps to meet regulatory standards. These steps can increase production costs and reduce overall yield, making it less efficient for large-scale use. Additionally, the use of immobilized enzymes can enhance stability and reusability, reducing costs and improving efficiency. For example, α-L-fucosidase from *Thermotoga maritima* immobilized on Eupergit® CM, efficiently synthesized 2′-FL (Guzmán-Rodríguez et al., 2021). Immobilized β-*N*-acetyl-hexosaminidase from *Bifidobacterium bifidum* on Cu^2+^-agarose displayed double the specific activity of its free form for lacto-*N*-triose II synthesis (Ruzic et al., 2020), and β-galactosidase from *Bacillus circulans* immobilized on silica via a cross-linked layer-by-layer technique produced 2–3 times more LacNAc (Karimi et al., 2020). Effective purification methods are essential to achieve high-purity HMOs and techniques such as chromatography and membrane filtration can be optimized for the efficient separation and purification of HMOs from reaction mixtures.

Recent advancements in biotechnology have enabled the production of human milk oligosaccharides (HMOs) through the overexpression of glycosyltransferases and other enzymes in engineered systems. For example, α1,2-fucosyltransferase FutBc from *Bacillus cereus* was utilized for the production of 2′-FL. The *futBC* genes, along with *LAC12*, were introduced into *S. cerevisiae*, which produced 27 g/L of 2′-FL from lactose and sucrose (Xu et al., 2021). Companies such as Jennewein Biotechnologie, Inbiose, and Abbott Laboratories have patented innovative approaches for HMO synthesis using genetically modified bacteria, such as *E. coli*, transgenic non-human mammals and engineered cell lines (Table 7). These patents cover the production of various HMOs, including 2′-FL, 3-FL, LNT, and sialylated oligosaccharides, highlighting their potential applications in infant formula and therapeutic products. Bacteria have been widely utilized as host systems, with companies such as Jennewein Biotechnologie GmbH producing sialylated oligosaccharides, 2′-fucosyllactose (2′-FL), and 3-fucosyllactose (3-FL) (patents US20210087599A1, EP2927316A1, and WO2010142305A1). Similarly, Glycosyn LLC has focused on generating fucosylated oligosaccharides in bacteria, with patents US9970018B2 and US10815511B2. Yeast-based systems have also proven effective, as demonstrated by Amyris Inc., which developed yeast strains to produce 2′-FL (US10519475B1). Beyond microbial platforms, transgenic non-human mammals have been employed by Abbott Laboratories for the production of HMOs, including 2′-FL and complex glycoproteins (US5750176A, US6204431B1, and US5700671A). Additionally, neutral, non-fucosylated HMOs have been synthesized using genetically engineered cells, including bacteria, yeast, and animal cells, by Inbiose (US12077788B2).

**TABLE 7:** Patents for human milk oligosaccharide production.

Synthesis method	Cell system used	HMOs produced	Company/Applicants	Patent number
Chemical	n/a	Double branched HMOs	University of Shandong	CN116217633A
Enzymatic	n/a	Fucosylated oligosaccharides	Debut Biotechnology Inc.	WO2025015168A1
Enzymatic	n/a	DSLNT	Shandong maternal and child health hospital	CN119040414A
Enzymatic	n/a	Not disclosed	Glycom A/S	US11214588B2
Chemoenzymatic	n/a	LNH	Ocean University of China	CN118325989A
Chemoenzymatic	n/a	LNnH	Ocean University of China	CN118166049A
Microbial Fermentation	Genetically engineered bacteria	Sialylated oligosaccharides	Jennewein Biotechnologie GmbH	US20210087599A1
Microbial Fermentation	Genetically engineered bacteria	2′-FL	Jennewein Biotechnologie GmbH	EP2927316A1
Microbial Fermentation	Genetically engineered bacteria	2′-FL and 3-FL	Jennewein Biotechnologie GmbH	WO2010142305A1
Microbial Fermentation	Transgenic non-human mammals	2′-FL	Abbott Laboratories	US5750176A
Microbial Fermentation	Transgenic non-human mammals	Various oligosaccharides and glycoprotein	Abbott Laboratories	US6204431B1 US5700671A
Microbial Fermentation	Genetically engineered cell (bacterium, yeast or animal)	Neutral, non-fucosylated HMOs	Inbiose	US12077788B2
Microbial Fermentation	Genetically engineered yeast	2′-FL	Amyris Inc.	US10519475B1
Microbial Fermentation	Genetically engineered bacteria	Fucosylated oligosaccharides	Glycosyn LLC	US9970018B2 US10815511B2

### 4.5 *In silico* advances in glycosyltransferases and HMO synthesis

**FIGURE 6:**
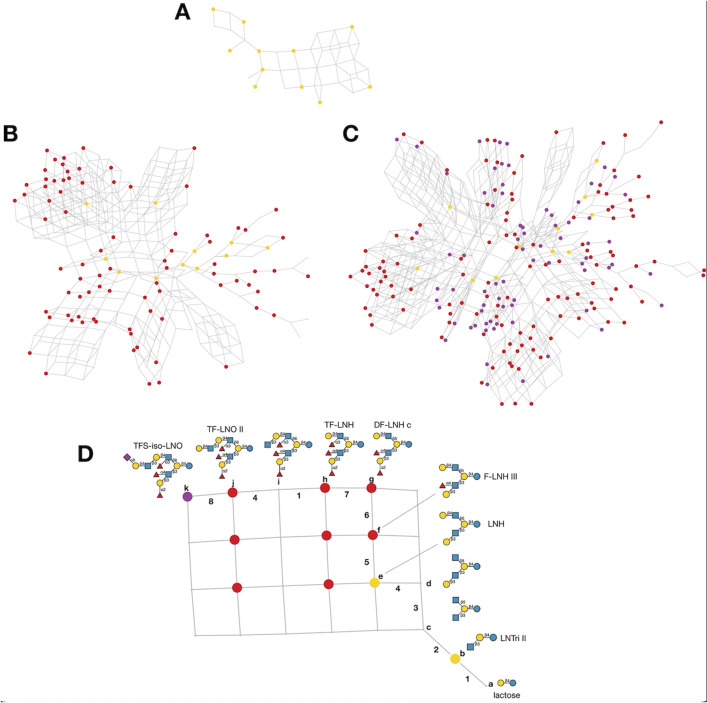
Networks of HMO biosynthesis simulated using Glycologue. Minimal reaction networks generated using the HMO-Glycologue enzyme simulator (McDonald et al., 2016b). Larger nodes represent experimentally characterised structures, coloured according to the type: yellow (core), red (neutral) or purple (acidic), while smaller nodes represent the intermediates predicted by the simulator. **(A)** Core structures, afucosylated and asialylated. **(B)** Neutral (fucosylated, asialylated) HMOs. **(C)** Acidic (sialylated) HMOs. **(D)** A proposed biosynthetic network of the acidic HMO, TFS-iso-LNO (Kitagawa et al., 1993; Urashima et al., 2018), showing the reactions catalysed by eight of the glycosyltransferase enzymes assumed to be active during lactation: (1) β3GnT (iGnT, EC 2.4.1.149); (2) dIGnT (EC 2.4.1.386); (3) β3GalT (EC 2.4.1.86); (4) β4GalT (EC 2.4.1.38); (5) α4FucT (EC 2.4.1.65); (6) α2FucT (type 1) (EC 2.4.1.69); (7) α3FucT (EC 2.4.1.152); (8) ST6Gal (EC 2.4.3.1). The sequence of enzyme activities (1,2,3,4,5,6,7,1,4,8) produces the path through nodes labelled a–k.

Recent advances in *in silico* modelling have revolutionized our understanding of glycosyltransferases and their application in HMO synthesis. Computational approaches now enable the prediction, simulation, and optimization of glycosylation pathways, significantly accelerating research and development in this field. The rapid advancements in glycobiology have been supported by the development of comprehensive databases and analytical tools, which facilitate the study of glycosylation patterns and enzyme functions critical for HMO biosynthesis (Artemenko et al., 2012). Additionally, computational approaches, such as knowledge-based systems, have proven effective in predicting glycosylation network behaviour and modelling the effects of enzyme knockouts on *O*-glycosylation, offering strategic insights for glycoengineering applications (McDonald et al., 2016b). Tools such as Rosetta (Leman et al., 2020) and AlphaFold (Jumper et al., 2021) have provided high-resolution models of these enzymes, facilitating the identification of active sites and key residues involved in substrate binding and catalysis. These insights are particularly valuable for engineering glycosyltransferases with enhanced activity, specificity, or stability, essential for efficient HMO production. Moreover, pathway analysis software, such as O-Glycologue, allows for the *in silico* reconstruction of glycosylation networks (McDonald and Davey, 2022; McDonald et al., 2010). This tool generates predictive models of oligosaccharide biosynthesis, enabling researchers to map out and optimize glycosyltransferase-mediated pathways for HMO synthesis (Figure 6; McDonald et al., 2022). By incorporating enzyme kinetics, substrate availability, and reaction conditions, these simulations help identify bottlenecks and propose modifications to improve yield and efficiency. The use of machine learning algorithms further enhances the predictive power of *in silico* studies. By analysing large datasets of glycosyltransferase sequences and activities, machine learning models can predict enzyme behaviour under different conditions and suggest mutations to tailor enzymes for specific HMOs (Yang et al., 2018; Taujale et al., 2021b). Coupled with molecular docking and molecular dynamics simulations, these models provide a comprehensive toolkit for rational enzyme design.

In the context of HMO synthesis, these *in silico* advancements could enable the design of highly efficient synthetic pathways. For example, computational approaches have been used to identify and engineer glycosyltransferases for increased turnover rates and reduced byproduct formation (Go et al., 2023; Yang et al., 2024), with the potential to facilitate scalable HMO production. For example, Rosetta enzyme design has been applied to engineer UGT76G1, resulting in enhanced thermostability and activity. Additionally, predictive models are being used to identify novel glycosyltransferases with unique capabilities, broadening the repertoire of enzymes available for synthetic biology applications.

### 4.6 Analytical techniques in HMO structure elucidation

Quantifying and profiling the diverse range of HMOs is crucial for understanding their role in infant nutrition and for the quality control of HMO production processes. Various analytical techniques are employed to detect different HMOs, determine their quantities, and profile their types. The analysis of HMOs often utilizes a separation method paired with a detection system that can deliver linear response curves to determine oligosaccharide concentrations. These methods ensure accurate identification and quantification of HMOs in both natural and industrially synthesized samples. Prior to using analytical technique for HMO quantification and elucidation, HMO samples are processed and isolated, be that from breast milk or alternative synthesis methods. These extractions include; solid phase extraction (McGuire et al., 2017), centrifugal fat separation (van Leeuwen et al., 2014), liquid extraction (De Leoz et al., 2013), ultrafiltration (Tonon et al., 2019), organic solvent precipitation (Galeotti et al., 2014), and gel filtration chromatography (Martín-Sosa et al., 2003). Comprehensive reviews detailing the analytical techniques used for HMO characterization are available elsewhere for further reference van Leeuwen (2019), Yan et al. (2017), and Niñonuevo and Lebrilla (2009).

#### 4.6.1 Chromatographic techniques

High-performance liquid chromatography (HPLC) is extensively used for separating and quantifying HMOs (Christensen et al., 2020). An advantage of HPLC is its ability to accurately quantify industrially synthesized HMOs of known structure, enabling precise monitoring of production yield and purity. Different HPLC modes, such as reverse-phase (RP-HPLC) (Josic and Kovac, 2010), normal-phase (NP-HPLC) (Liu et al., 2017), and hydrophilic interaction liquid chromatography (HILIC) (Tang et al., 2016), allow for the effective separation of HMOs based on their size, polarity, and charge. HPLC can be coupled with various detectors, including ultraviolet (UV), refractive index (RI), and fluorescence detectors (Nikolin et al., 2004), to quantify HMOs. HPLC combined with mass spectrometry (HPLC-MS), enhances the capability to both separate and identify HMOs in complex sample (Csernák et al., 2020). Fluorescent labelling involves tagging HMOs with fluorescent dyes, enhancing their detectability and quantifiability.

High-performance anion-exchange chromatography (HPAEC) coupled with pulsed amperometric detection (PAD) is a powerful technique for separating and quantifying HMOs based on their monosaccharide composition and charge (Mechelke et al., 2017). HPAEC-PAD is highly sensitive and can detect HMOs at very low concentrations. This method is particularly useful for analysing neutral and acidic oligosaccharides, providing detailed profiles of HMO mixtures (Gu et al., 2021).

#### 4.6.2 Electrophoretic techniques

Capillary electrophoresis (CE) separates HMOs based on their charge-to-mass ratio (Amézqueta et al., 2020) and offers high resolution and efficiency. Techniques such as capillary zone electrophoresis (CZE) and capillary gel electrophoresis (CGE) are employed to analyse HMOs. A big problem with CE is the fact that the migration of the HMOs is determined by this charge-to-mass ratio, which means that structural isomers will elute at the same time, such as 3′-SL and 6′-SL, 2′-FL and 3-FL, etc (van Leeuwen, 2019). Although there has been cases in which some structural isomers can be distinguished from each other in CE approaches (Galeotti et al., 2014). CE can be coupled with laser-induced fluorescence (LIF) detection for enhanced sensitivity and quantification. Additionally, CE-MS combines the high separation efficiency of CE with the detection capabilities of mass spectrometry, providing both qualitative and quantitative information on HMOs.

#### 4.6.3 Mass spectrometry techniques

Mass spectrometry is a vital tool for the identification and quantification of HMOs due to its sensitivity and specificity (Wu et al., 2017). This analytical technique identifies and quantifies substances by measuring the mass-to-charge ratio of ionized particles. Mass spectrometry enables the identification of unknown HMO structures by analysing their molecular weight and fragmentation patterns. Techniques such as Matrix-Assisted Laser Desorption/Ionization (MALDI-MS) and Electrospray Ionization (ESI-MS) are widely used (Porfirio et al., 2020). MALDI-MS is effective for the rapid profiling of HMOs in complex mixtures, providing a broad overview of their molecular weights and relative abundances. ESI-MS, often coupled with liquid chromatography (LC-ESI-MS), offers high sensitivity and can quantify HMOs with greater accuracy by providing detailed mass and fragmentation data, allowing for the discrimination of HMOs with similar masses (Ho et al., 2003).

#### 4.6.4 Nuclear magnetic resonance spectroscopy technique

Nuclear Magnetic Resonance (NMR) Spectroscopy is primarily used for structural elucidation (Kaliva et al., 2020), it can also quantify HMOs by integrating the signals corresponding to different oligosaccharides (Kortesniemi et al., 2022). Quantitative NMR (qNMR) provides accurate and non-destructive quantification of HMOs without the need for extensive sample preparation. NMR is particularly useful for analysing complex mixtures and providing absolute quantification of individual components (Bharti and Roy, 2012). NMR spectroscopy is uniquely suited for distinguishing stereoisomers of HMOs, particularly in differentiating α- vs. β-linked glycosidic bonds, which are challenging to resolve using mass spectrometry alone (Garádi et al., 2023). Therefore it is useful in analysis of HMO concentration in breast milk samples (van Leeuwen et al., 2014; Garádi et al., 2023; Praticò et al., 2014; Gómez-Gallego et al., 2018).

Accurate quantification and profiling of HMOs are essential for understanding their biological roles and for the quality control of HMO products. Techniques such as mass spectrometry, HPLC, capillary electrophoresis, HPAEC-PAD, fluorescent labelling, and NMR spectroscopy each offer unique advantages for the detection, quantification, and profiling of HMOs. By employing these advanced analytical methods, researchers and industry professionals can ensure the precise and comprehensive analysis of HMOs, supporting their application in infant nutrition and therapeutic products.

## 5 Future work

Investigating glycosyltransferases and their involvement in HMO biosynthesis opens new opportunities for advancing HMO production methods. By uncovering the intricate mechanisms through which these enzymes assemble complex oligosaccharides, researchers can identify key targets for optimizing both natural and synthetic production systems. Future work should focus on refining enzyme engineering approaches to enhance efficiency, specificity, and yield, while also exploring how these modifications might impact the biological activity of HMOs. Such advancements have the potential to not only improve commercial scalability but also deepen our understanding of the therapeutic applications of HMOs in immune modulation and beyond. By improving our ability to produce HMOs commercially, these insights pave the way for exciting advancements in both research and industry, while emphasizing the need for continued exploration in this field.

While much research has focused on the development of methods to synthesize simple HMOs, such as 2′-FL and 3′-SL, the synthesis of branched antennary HMOs remains critical yet understudied. These branched HMOs, including symmetrically branched structures such as LNnH and asymmetrically branched structures such as LNH, exhibit structural complexity and unique glycan motifs that contribute to their roles in modulating the infant gut microbiota and shaping immune responses. Despite their biological significance, branched HMOs are naturally present in low abundance, and their isolation from human milk is labour-intensive and cost-prohibitive. Synthetic approaches are essential to produce sufficient quantities for detailed functional studies and potential therapeutic applications. Chemical and enzymatic synthesis strategies have shown promise in constructing branched HMOs with high precision (Prudden et al., 2017; Bandara et al., 2020; Xiao et al., 2016), however, these approaches face challenges such as achieving regioselectivity, stereoselectivity, and efficiently utilizing glycosyltransferase enzymes to catalyse complex glycosidic linkages. Advances in chemoenzymatic synthesis, including the engineering of glycosyltransferases and the optimization of nucleotide sugar donors, have begun to address these obstacles. The ability to synthesize branched HMOs not only provides a means to explore their unique biological functions but also offers the potential to unlock their therapeutic benefits.

Future research should focus on refining the synthetic production of HMOs to expand their therapeutic applications beyond infant nutrition. Optimising glycosyltransferase activity and biosynthetic pathways could enhance the scalability and efficiency of HMO synthesis, paving the way for novel applications as currently their most obvious application is in infant formula enhancement, where artificially synthesized HMOs, such as 2′-FL and LNT, are added to mimic the beneficial effects of human milk (Vandenplas et al., 2018). Further studies are needed to explore the functional properties of HMOs and their glycosylated derivatives in modulating gut microbiota, immune responses, and disease prevention. Another promising area is antimicrobial therapeutics, as HMOs can act as decoy receptors, preventing pathogens such as *Norovirus* (Koromyslova et al., 2017) and *Helicobacter pylori* (Gustafsson et al., 2006; Simon et al., 1997) from binding to host cells, reducing infection risks. In pathogen receptor research, studying glycosyltransferase-modified glycans helps identify sugar-binding interactions used by viruses and bacteria to infect cells, paving the way for novel antiviral and antibacterial strategies. Companies specializing in carbohydrate-based drug discovery, such as GlycoMimetics (Smith and Bertozzi, 2021) and Databases such as SugarBind (Mariethoz et al., 2015) and GlyGen (York et al., 2020) that focus on human glycan structures binding specificities, glycosylation data, and structural annotations, are leveraging this knowledge to develop glycan-inspired therapeutics targeting inflammatory diseases, cancer, and infectious diseases. HMOs have exciting potential for treating autoimmune and inflammatory diseases such as inflammatory bowel disease (IBD) (Iribarren et al., 2020; Palsson et al., 2020) and rheumatoid arthritis (RA) (Kim et al., 2022). Their immunomodulatory properties, particularly their ability to shape immune cell and cytokine responses (Slater et al., 2024), make them promising therapeutic candidates.

The role of glycosyltransferases in the biosynthesis of HMOs is proving indispensable for replicating these compounds for commercial use, especially in the enhancement and fortification of infant formula. However, several challenges must be addressed to optimize this approach for commercial production including the technical challenges in glycosyltransferase characterization and engineering, strategies to enhance HMO yield, diversity, and purity, and future prospects for advancing glycosyltransferase-based synthesis.

## 6 Conclusion

The integration of computational tools, including molecular modelling and machine learning, can accelerate the characterization and engineering of glycosyltransferases. Predictive models can identify key residues involved in substrate binding and catalysis, guiding rational enzyme design. Synthetic biology offers innovative solutions for HMO synthesis by enabling the construction of synthetic pathways and the development of novel microbial platforms. The design of synthetic operons and the use of modular genetic elements can enhance the flexibility and efficiency of HMO production systems. The exploration of natural sources, including diverse microbial and plant species, can lead to the discovery of novel glycosyltransferases with unique specificities and catalytic properties. Metagenomic and bioinformatic approaches can aid in identifying and characterizing new enzymes for HMO synthesis. Future advancements should focus on sustainable and cost-effective production methods. This includes the development of renewable feedstocks, the use of low-cost substrates, and the implementation of environmentally friendly processes. Reducing the environmental footprint of HMO production is essential for long-term viability. The glycosyltransferase-mediated synthesis of HMOs presents both significant challenges and exciting opportunities. Addressing the technical challenges in enzyme characterization and engineering, optimizing production strategies, and leveraging future advancements in computational and synthetic biology are critical for advancing this field. By overcoming these obstacles, the production of HMOs can be scaled up to meet growing demands, providing valuable benefits for infant nutrition and therapeutic applications.
